# Burden of chronic obstructive pulmonary disease and its attributable risk factors in China from 1990 to 2021, with projections to 2050: an analysis of data from the Global Burden of Disease study 2021

**DOI:** 10.3389/fmed.2025.1644022

**Published:** 2025-09-03

**Authors:** Min Liu, Shuoshuo Wei, Xin Yang, Zhuoyuan Lu, Wanwan Zhang, Emmanuel Mensah, Lei Zha, Yun Zhou

**Affiliations:** ^1^Department of Graduate School of Bengbu Medical University, Bengbu Medical University, Bengbu, Anhui, China; ^2^Department of Pulmonary and Critical Care Medicine, The Second People's Hospital of Wuhu, Wuhu, Anhui, China; ^3^Department of Pulmonary and Critical Care Medicine, The First Affiliated Hospital of Wannan Medical College (Yijishan Hospital of Wannan Medical College), Wuhu, Anhui, China; ^4^School of Information Science and Engineering, Lanzhou University, Lanzhou, Gansu, China

**Keywords:** chronic obstructive pulmonary disease, burden of disease, epidemiology, risk factors, Global Burden of Disease, China

## Abstract

**Background:**

Chronic Obstructive Pulmonary Disease (COPD) has become the third leading cause of death worldwide. This disease not only results in high mortality rates but also triggers substantial medical expenditures, significant loss of labor productivity, and a marked decline in patients’ quality of life. Despite its severity, COPD is a preventable condition and has now emerged as a significant public health burden that cannot be overlooked. This study aimed to assess the burden of COPD and its attributable risk factors from 1990 to 2021 and to project trends through 2050, to provide an evidence basis for the development of a comprehensive COPD prevention and treatment strategy in China.

**Methods:**

Data were extracted from the Global Burden of Disease (GBD) 2021 study, including COPD-related incidence, prevalence, mortality, and disability-adjusted life years (DALYs). Age-standardized rates (ASRs) were calculated, and temporal trends were analyzed using estimated annual percentage change (EAPC) through linear regression modeling. A Bayesian age-period-cohort (BAPC) model was used to forecast trends up to 2050.

**Results:**

In 2021, China recorded 50.6 million prevalent COPD cases, 4.4 million incident cases, 1.29 million deaths, and 23.6 million DALYs. Between 1990 and 2021, the age-standardized incidence rate (ASIR) declined from 271.2 to 215.6 per 100,000 population; the age-standardized prevalence rate (ASPR) from 2,761.8 to 2,499.4 per 100,000; age-standardized mortality rate (ASMR) from 231.8 to 73.2 per 100,000; and age-standardized DALYs (ASDALYs) from 3,852.6 to 1,227.7 per 100,000. These reflect relative reductions of 20.5, 9.5, 68.4, and 68.1%, respectively.

**Conclusion:**

Despite substantial reductions in the COPD burden over the past three decades, the disease continues to pose a major health challenge in China, particularly among the aging population. Projections to 2050 indicate continued, though uneven, declines. These findings underscore the urgent need for strengthened diagnostic capacity, risk-targeted prevention efforts, and more effective long-term management strategies tailored to China’s aging population.

## 1 Introduction

### 1.1 Background on chronic obstructive pulmonary disease (COPD) and its public health significance

Chronic obstructive pulmonary disease (COPD) represents a major public health and economic challenge both globally and in China (1). It is a heterogeneous respiratory condition characterized by persistent airflow limitation, primarily resulting from small airway disease (e.g., obstructive bronchiolitis) and parenchymal destruction (emphysema) (2, 3). Chronic inflammation drives structural remodeling of the lungs, leading to airway narrowing, mucociliary dysfunction, and hallmark symptoms such as progressive dyspnea, chronic cough, sputum production, and chest tightness (4, 5). In advanced stages, patients may also experience fatigue, weight loss, muscle wasting, and anorexia, compounding the individual and societal burden (6–8).

### 1.2 COPD burden in China: patterns and trends from 1990 to 2019

Between 1990 and 2019, COPD consistently ranked among the leading causes of death worldwide, including in China (1). In 2019, it was the third leading cause of death globally, accounting for approximately 212.3 million prevalent cases, 3.3 million deaths, and 74.4 million disability-adjusted life years (DALYs). According to the Global Burden of Disease (GBD) 2019 study, global age-standardized prevalence, mortality, and DALY rates were 2,638.2, 42.5, and 926.1 per 100,000 population, respectively (9). Notably, China reported the highest national COPD mortality rate, underscoring the urgent need for targeted national strategies.

COPD also worsens outcomes in comorbid conditions such as cardiovascular disease, cancer, and infectious diseases (10). Comorbidities including COVID-19, tuberculosis, and asthma accelerate disease progression and mortality, placing considerable emotional and financial stress on affected families (11–13). These interactions complicate clinical management and amplify the broader societal and economic impact of COPD (14).

### 1.3 Economic burden of COPD

Beyond clinical consequences, COPD imposes a substantial economic burden. In China, limited insurance coverage and high out-of-pocket costs often restrict access to diagnosis and treatment, particularly among low-income and elderly individuals without stable income or pension support (15, 16). Globally, the economic impact of COPD is projected to reach USD 43.26 trillion between 2020 and 2050 (17, 18), with China alone estimated to incur USD 1.4 trillion in losses during the same period (19, 20). These constraints contribute to underdiagnosis, poor treatment adherence, and reduced workforce productivity.

### 1.4 Core risk factors for COPD

Environmental and behavioral risk factors remain central to the development of chronic obstructive pulmonary disease (COPD), which is closely associated with modifiable factors including smoking, environmental particulate matter, occupational exposures (e.g., dust, gases), secondhand smoke, and household air pollution (21). Notably, smoking as the primary risk factor contributes to approximately 46% of COPD cases (22), while air pollution (ambient and household) represents the leading contributor to COPD-related disability-adjusted life years (DALYs) (9, 23). In China, this risk distribution exhibits urban–rural disparities: severe indoor air pollution from biomass fuel use for cooking/heating dominates in rural areas, whereas urban residents face greater threats from industrial emissions and smoking (24). Risk exposure demonstrates significant population heterogeneity—males face elevated risks due to high smoking rates and occupational/environmental toxins (e.g., asbestos, soot) (24), particularly among those aged ≥60 years, smokers, and adults >40 years (5, 25); females, low-income households, occupationally exposed individuals, and those with obesity experience greater lung function decline and COPD incidence when exposed to air pollutants (26); and socioeconomically disadvantaged groups in low- and middle-income countries (LMICs) suffer compounded effects of air pollution and occupational exposures, driving higher COPD and asthma prevalence (27). Amid population aging and persistently high smoking rates, global COPD prevalence is projected to increase by 23% by 2050, disproportionately affecting these vulnerable populations (28, 29). Cross-disease evidence further reveals: air pollution constitutes the foremost avoidable risk for asthma exacerbations, while smoking is the dominant risk factor for COPD and lower respiratory diseases, with impoverished groups exhibiting universal susceptibility to respiratory diseases due to multi-source exposures and limited healthcare access (30). This underscores the urgency for targeted interventions—including tobacco control, clean energy transitions, occupational protections, and healthcare equity—alongside addressing climate change’s long-term threats to respiratory health (27).

### 1.5 Infrastructure limitations for COPD diagnosis and treatment

The disease burden is further exacerbated by limited diagnostic and treatment infrastructure. Only 11.4% of county-level hospitals in China reportedly provide bronchoscopy services, with significant regional disparities. For instance, bronchoscopy use has increased 3.4-fold in low-GDP provinces such as Hunan compared to a 5.8-fold increase in more developed areas like Shanghai (2002–2017) (31, 32). In rural Hunan, only 47.9% of hospitals offer diagnostic bronchoscopy, and just 6.3% provide therapeutic procedures (33). These disparities hinder early diagnosis and effective disease management, particularly in underserved regions.

### 1.6 Limitations of existing research

Although prior studies have assessed COPD burden using GBD 2017 and 2019 data, none have incorporated the latest GBD 2021 dataset to evaluate evolving trends post-2020. Existing analyses often lack consideration of demographic shifts, emerging risk factor dynamics, and long-term projections, critical for policy planning. Furthermore, few studies have applied GBD’s comparative risk assessment framework to quantify modifiable risk factors, and none have offered baseline estimates ahead of COPD’s inclusion in China’s Basic Public Health Service Program in 2024.

### 1.7 Objectives and significance of this study

Given China’s ongoing demographic transition—characterized by a vast, rapidly aging population and increasing demands for equitable healthcare—understanding the long-term epidemiological dynamics and key drivers of chronic obstructive pulmonary disease (COPD) has become imperative for evidence-based health policy formulation. The current absence of recent, forward-looking evaluations with explicit policy relevance consequently constrains strategic resource allocation and impedes informed decision-making in respiratory health management. To address these critical evidence gaps, this study leverages comprehensive Global Burden of Disease (GBD) 2021 data to conduct a systematic assessment of COPD’s spatiotemporal burden and attributable risk factors across China from 1990 to 2021, with Bayesian modeling projections extending to 2050. Ultimately, these evidence-based findings aim to catalyze the development of targeted, population-stratified public health interventions tailored to China’s evolving healthcare landscape.

## 2 Methods

### 2.1 Data sources

Based on the Global Burden of Disease (GBD) 2021 study data maintained by the Institute for Health Metrics and Evaluation (IHME), this research utilized a comprehensive dataset synthesizing diverse sources—including censuses, surveys, vital registration, disease registries, and environmental monitoring—to estimate the global burden of 371 diseases/injuries and 88 risk factors across 204 countries/territories from 1990 to 2021 (mortality: 1980–2021), with China’s COPD burden data (incidence, prevalence, mortality, DALYs) specifically extracted from the Global Health Data Exchange (GHDx: http://ghdx.healthdata.org/gbd-results-tool). The GBD methodology employs three core modeling tools (Cause of Death Ensemble model/CODEm, Spatiotemporal Gaussian Process Regression/ST-GPR, and Disease Model–Bayesian Meta-Regression/DisMod-MR) to generate estimates for six key metrics—incidence, prevalence, mortality, years of life lost (YLLs), years lived with disability (YLDs), and disability-adjusted life years (DALYs)—spanning 23 age groups (0 to ≥95 years), both sexes, and 204 geographical units. Key analytical steps included calculating age-standardized rates (ASRs) using the GBD reference population, determining estimated annual percentage changes (EAPC) to quantify temporal trends, and deriving 95% uncertainty intervals (UIs) from 1,000 posterior draws (reported as 2.5th–97.5th percentiles) (34, 35).

### 2.2 Case definition and disease classification

COPD was defined according to the Global Initiative for Chronic Obstructive Lung Disease (GOLD) criteria, characterized by a post-bronchodilator forced expiratory volume in 1 s (FEV₁) to forced vital capacity (FVC) ratio of less than 0.7 (2). COPD cases were identified using International Classification of Diseases, 10th Revision (ICD-10) codes (e.g. J41, J42, J43, J44, and J47).

### 2.3 Statistical analysis

Descriptive analyses evaluated the COPD burden across 21 age groups (1 to ≥95 years, in 5-year intervals) and by gender. Metrics included ASIR, ASMR and ASDALYs.

To analyze trends in age-standardized rates (ASRs) of COPD incidence, prevalence, mortality, and DALYs, we employed the Estimated Annual Percentage Change (EAPC) method. This approach involves the following regression model (36): In (ASR) = *α* + *β*X + *ε*. In this equation, ln (ASR) represents the natural logarithm of the age-standardized rate, *Χ* denotes the calendar year, α is the intercept on the y-axis, and β is the slope indicating temporal trends. Any error in the model is represented by ε. Calendar year was used as the explanatory variable X when calculating the EAPC, and the logarithm (ln [ASR]) based on the natural number of the ASR was used as the dependent variable Y to fit the data to the regression line y = a + bx + ε. Subsequently, we used the fitted regression line parameter b to calculate the EAPC as follows: EAPC = 100 × [exp (β) − 1]. The EAPC can only be calculated when ASR changes remain stable throughout the observation period. Statistical hypothesis testing is required to assess the calculated EAPC to exclude the influence of random factors. The hypothesis test for the EAPC is equivalent to the hypothesis test for the slope of a fitted line, i.e., if the slope of the line is statistically significant, the EAPC is considered effective. The hypothesis test for EAPC is a *t* test of slope *b* of the fitted line: *tb* = *b*/sb (*b* is the slope of the line and sb is the SE of slope b), and the degrees of freedom, V, is the number of calendar years minus 2. Because of the influence of the SE of slope b on the slope of the fitted line and EAPC, the 95% confidence intervals (CIs) of the EAPC need to be calculated as follows:

In(ASR)=a+bx+ε;

EAPC=100×(exp(β)−1)

If the EAPC estimate and its 95% CI lower bound are both greater than 0, ASRs are considered to be increasing; if the upper bound is less than 0, ASRs are considered to be decreasing; otherwise, ASRs are deemed stable over time. (36, 37).

To disentangle the effects of age, time period, and birth cohort on COPD mortality, an age-period-cohort (APC) model was applied. This model considers the complex interplay of biological, environmental, and social factors influencing disease trends over time, extending traditional epidemiological analyses (38).

The Bayesian age-period-cohort (BAPC) model was applied to predict COPD incidence, prevalence, mortality, and DALYs from 2022 to 2050. The BAPC model extends the generalized linear model (GLM) framework within a Bayesian context, dynamically integrating age, period, and cohort effects. These effects exhibit continuous evolution over time and are smoothed via a second-order random walk, enhancing posterior probability prediction accuracy. A key advantage is its implementation of Integrated Nested Laplace Approximation (INLA) for approximating marginal posterior distributions. This approach circumvents challenges—including mixing and convergence issues inherent in Markov Chain Monte Carlo techniques—while preserving computational efficiency. The model’s flexibility and robustness in analyzing time series data render it particularly suitable for long-term disease burden forecasting. Given its comprehensive temporal trend capture, the BAPC model has undergone extensive validation and application in epidemiological research, especially for age-structured population data and complex cohort effects (39, 40).

Statistical analyses and data visualizations were conducted using R (version 4.4.1). Descriptive statistics were generated for all key vari ables. Data downloaded from the GBD study 2021 such as numbers, rate and ASR of indicators were presented as means with 95% UI, while results calculated by our study such as EAPC and BAPC predicted ASRs were presented as means with 95% confidence intervals (CI). A *p*-value < 0.05 was considered statistically significant for trend analyses.

### 2.4 Risk factors

This study assessed established COPD risk factors including tobacco smoking, ambient particulate matter pollution, occupational exposure to particulates and gases, household air pollution from solid fuels, secondhand smoke, ambient ozone pollution, and extreme temperatures. The attributable fractions of DALYs for each risk factor were calculated based on prior literature and GBD data (34).

## 3 Results

### 3.1 Overall burden of COPD in China, 1990–2021

The overall burden of chronic obstructive pulmonary disease (COPD) in China demonstrated a marked decline from 1990 to 2021 across multiple metrics. In 1990, COPD ranked as the second leading cause of death based on ASMR; by 2021, it had become the third leading cause, accounting for 10.99% of all deaths. In that year, China reported 50.5 million prevalent cases of COPD, 4.4 million incident cases, 1.3 million deaths, and 23.6 million disability-adjusted life years (DALYs). Male deaths comprised 52% of the total, while females accounted for 48%.

Between 1990 and 2021, the ASIR decreased from 271.22 to 215.62 per 100,000 population (estimated annual percentage change (EAPC): −0.843; 95% confidence interval (CI): −0.878 to −0.808) (Table 1). The ASPR declined from 2,761.81 to 2,499.37 per 100,000 (EAPC: –0.333; 95% CI: −0.375 to −0.291) (Supplementary Table S1). Moreover, the ASMR dropped from 231.78 to 73.23 per 100,000 (EAPC: –4.250; 95% CI: −4.483 to −4.017), and the ASDALYs fell significantly from 3,852.57 to 1,227.66 per 100,000 (EAPC: –4.186; 95% CI: −4.382 to −3.990) (Supplementary Table S2, S3).

**Table 1:** Incidence cases and rates of chronic obstructive pulmonary disease (COPD) in 1990 and 2021, and the time trend from 1990 to 2021.

Incidence (95%UI)					
Age	1990		2021		EAPC, 95%CI (1990–2021)
	Number	rate per 100,000	Number	rate per 100,000	
All age	2160980.886 (1992125.013, 2311357.491)	183.685 (169.333, 196.468)	4434437.769 (4008615.310, 4860417.981)	311.682 (281.752, 341.622)	1.656 (1.600, 1.712)
age-standardized		271.222 (251.664, 288.622)		215.620 (198.000, 234.903)	−0.843 (−0.878, −0.808)
15–19 years	33314.176 (24835.879, 40015.136)	26.301 (19.607, 31.591)	14306.380 (9787.752, 19393.386)	19.159 (13.108, 25.971)	−1.160 (−1.273, −1.047)
20–24 years	47875.596 (35433.222, 58550.610)	36.269 (26.843, 44.356)	19113.758 (13037.609, 26165.056)	26.121 (17.817, 35.757)	−1.276 (−1.393, −1.159)
25–29 years	42136.295 (30642.539, 49720.889)	38.344 (27.885, 45.246)	26370.268 (17287.509, 34668.962)	30.492 (19.990, 40.088)	−0.925 (−1.022, −0.827)
30–34 years	34332.546 (25575.285, 40803.365)	38.906 (28.982, 46.239)	40721.808 (27787.706, 53141.572)	33.612 (22.936, 43.863)	−0.582 (−0.648, −0.516)
35–39 years	37914.085 (29761.345, 43243.321)	41.509 (32.583, 47.344)	40858.986 (30163.733, 49120.058)	38.560 (28.466, 46.356)	−0.273 (−0.298, −0.248)
40–44 years	87978.761 (56193.697, 124464.513)	131.127 (83.753, 185.507)	80836.760 (54152.729, 113342.933)	88.314 (59.162, 123.826)	−1.426 (−1.544, −1.309)
45–49 years	150358.740 (111225.003, 189265.627)	291.286 (215.473, 366.659)	212076.282 (148430.694, 290821.304)	192.235 (134.544, 263.612)	−1.507 (−1.583, −1.431)
50–54 years	179168.726 (141761.429, 210140.754)	375.530 (297.126, 440.446)	345489.392 (253309.751, 442322.424)	285.861 (209.591, 365.982)	−0.993 (−1.034, −0.952)
55–59 years	175879.798 (149355.959, 194532.180)	405.542 (344.383, 448.550)	392759.513 (316167.821, 463837.360)	357.241 (287.576, 421.891)	−0.452 (−0.476, −0.427)
60–64 years	275863.615 (201198.502, 351153.780)	780.655 (569.363, 993.715)	414650.359 (310698.089, 545862.356)	567.974 (425.584, 747.704)	−1.089 (−1.138, −1.039)
65–69 years	350317.661 (272303.602, 408325.106)	1284.070 (998.114, 1496.694)	729655.578 (525901.722, 920072.868)	951.267 (685.629, 1199.519)	−1.024 (−1.075, −0.973)
70–74 years	319606.954 (255822.084, 363905.540)	1698.442 (1359.479, 1933.852)	736483.531 (546471.966, 922922.541)	1381.862 (1025.344, 1731.678)	−0.728 (−0.787, −0.668)
75–79 years	224712.963 (185049.571, 250663.045)	1974.510 (1625.995, 2202.528)	574853.581 (451571.691, 670868.145)	1735.725 (1363.485, 2025.633)	−0.507 (−0.557, −0.456)
80–84 years	134991.905 (109851.963, 150215.170)	2548.399 (2073.803, 2835.786)	446916.235 (358048.971, 522564.184)	2258.081 (1809.072, 2640.299)	−0.529 (−0.576, −0.482)
85–89 years	52641.002 (44697.888, 58146.379)	3120.659 (2649.776, 3447.028)	251226.919 (202535.756, 289400.249)	2637.345 (2126.192, 3038.083)	−0.703 (−0.758, −0.649)
90–94 years	11824.712 (9542.234, 13663.875)	3853.931 (3110.022, 4453.355)	86624.538 (68365.354, 105078.031)	2954.465 (2331.707, 3583.850)	−1.064 (−1.130, −0.997)
95 + years	2063.351 (1446.011, 2541.557)	5095.689 (3571.095, 6276.675)	21493.880 (14048.630, 28835.852)	3363.156 (2198.195, 4511.958)	−1.582 (−1.673, −1.491)
Sex	Number	ASR* per 100, 000	Number	ASR* per 100, 000	EAPC
Female	1114590.105 (1028463.610, 1188400.257)	269.849 (251.760, 286.112)	2207589.699 (1987781.195, 2431561.134)	204.657 (185.881, 224.539)	−0.956 (−1.055, −0.856)
Male	1046390.781 (957619.019, 1124673.577)	274.178 (252.572, 293.139)	2226848.071 (2022286.573, 2432575.100)	229.215 (211.290, 246.901)	−0.708 (−0.797, −0.618)

*ASR, age-standardized rates; EAPC, estimated annual percentage change.

### 3.2 Gender-specific trends in COPD burden in China, 1990–2021

The burden of COPD remained consistently higher among males across all indicators; however, both sexes experienced steady declines over the study period. Among males, the ASIR decreased from 274.18 to 229.22 per 100,000 (EAPC: –0.708; 95% CI: −0.797 to −0.618), while in females it declined from 269.85 to 204.66 per 100,000 (EAPC: –0.956; 95% CI: −1.055 to −0.856) (Table 1). The ASPR in males fell from 2,638.55 to 2,479.43 per 100,000 (EAPC: –0.335; 95% CI: −0.497 to −0.173), while in females it declined from 2,854.45 to 2,492.11 per 100,000 (EAPC: –0.349; 95% CI: −0.443 to −0.256) (Supplementary Table S1).

The ASMR among males declined from 284.57 to 105.73 per 100,000 (EAPC: –3.571; 95% CI: −3.794 to −3.347), and from 199.34 to 52.73 per 100,000 in females (EAPC: –5.002; 95% CI: −5.284 to −4.719) (Supplementary Table S2). Correspondingly, DALYs in males decreased from 4,551.32 to 1,063.16 per 100,000 (EAPC: –3.737; 95% CI: −3.919 to −3.556), while in females they fell from 3,358.07 to 963.62 per 100,000 (EAPC: –4.671; 95% CI: −4.909 to −4.433) (Supplementary Table S3).

### 3.3 Age- and gender-specific trends of COPD burden in China, 1990–2021

**Figure 1:**
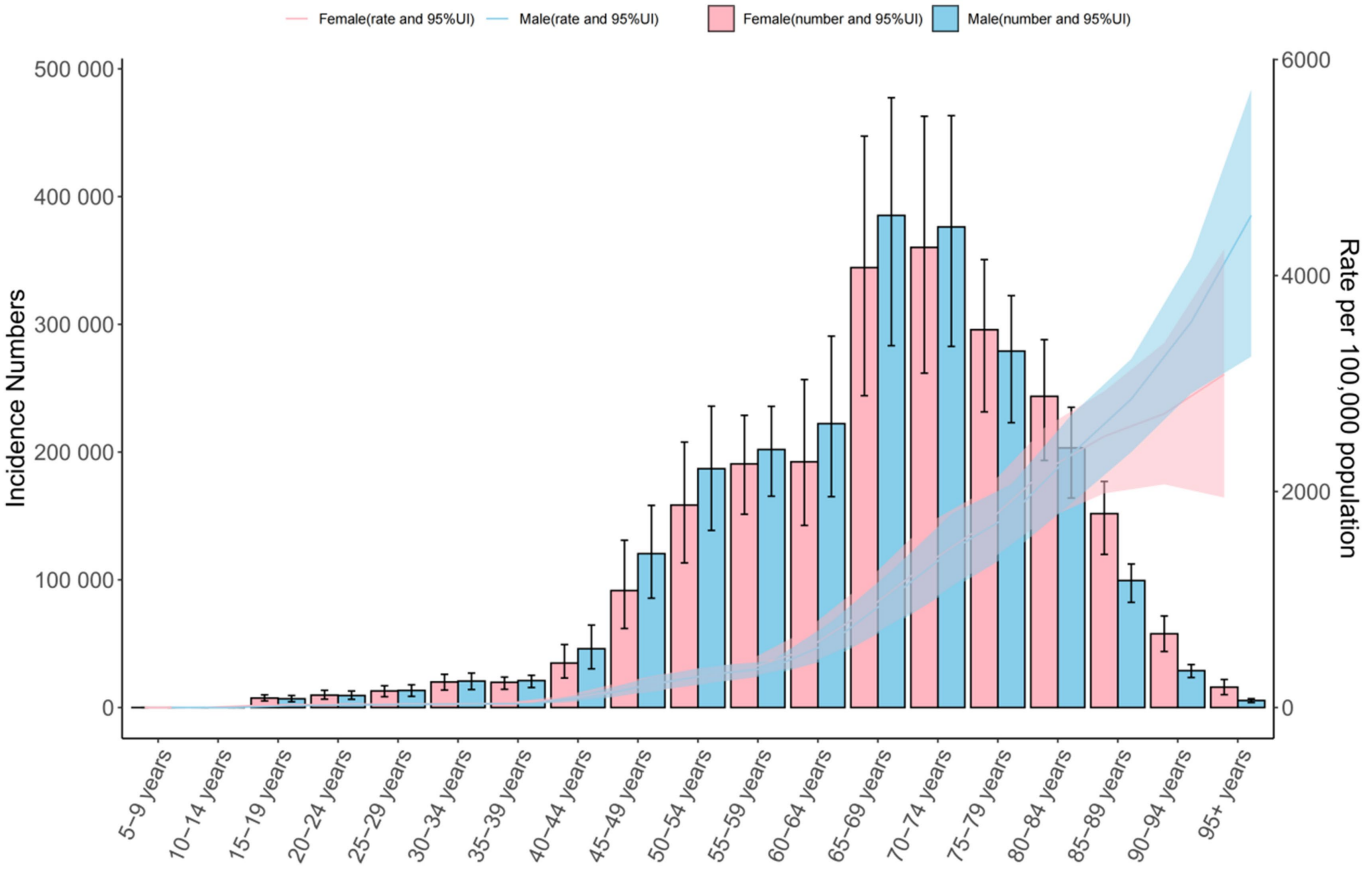
Global number of incident cases and incidence rate per 100,000 population of chronic obstructive pulmonary disease by age and sex in 2021. The lines represent the number of incident cases for males and females, 95%UI.

In 2021, COPD prevalence increased with age, beginning from the 15–19 age group and peaking among those aged ≥95 years. The highest number of prevalent cases was recorded in the 70–74 age group. Notably, prevalence was higher in males up to age 74, after which it became higher in females (Figure 1). COPD incidence exhibited a similar pattern, with the peak incidence occurring in the ≥95 age group and the largest number of new cases in the 70–74 age group. As with prevalence, incidence rates were higher in males before age 74 and higher in females thereafter.

Mortality rates increased with age, peaking in the ≥95 age group, though the highest absolute number of deaths occurred in the 80–84 age group. Across all age groups, mortality rates were consistently higher in males. DALY rates also rose with age for both sexes. In males, DALYs peaked in the 90–94 age group and then declined, while in females, rates continued to increase into the ≥95 age group. Across all age groups under 90, DALY rates were higher in males. The highest total DALYs occurred in the 75–79 age group for both sexes (Figure 1; Supplementary Figures S1–S3).

### 3.4 Age-period-cohort (APC) model of COPD mortality in China, 1990–2021

**Figure 2:**
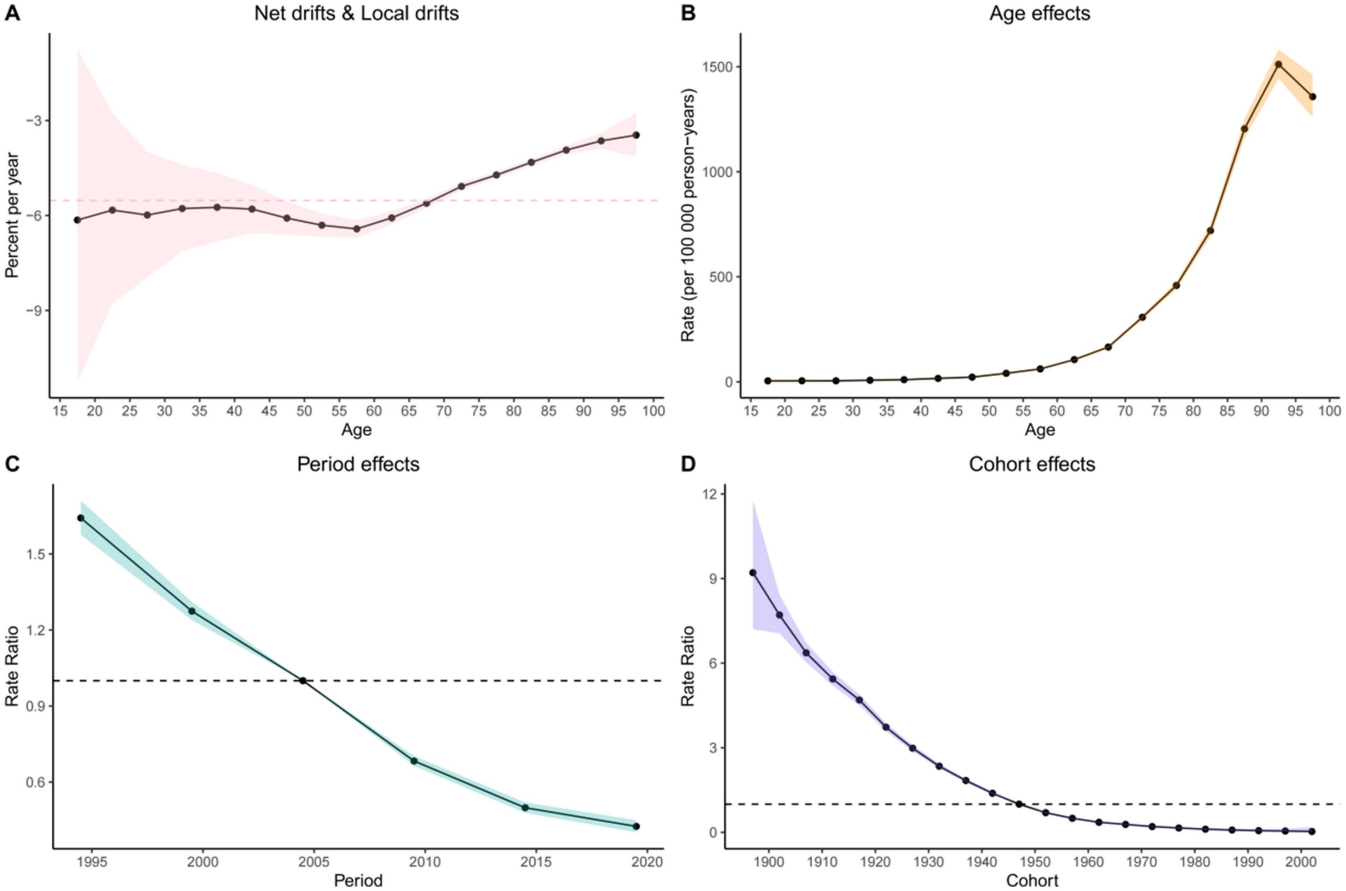
Visualization of the Bayesian age-period-cohort analysis model for COPD mortality in the Chinese population from 1990 to 2021. **(A)** Net drifts and local drifts on mortality relative risk. **(B)** Age effects on mortality relative risk; **(C)** Period effects on mortality relative risk; **(D)** Cohort effects on mortality relative risk.

The age-period-cohort (APC) model revealed a consistent decline in COPD mortality risk over time. From 1990 to 2021, the net drift, indicating the overall annual percentage change in ASMR, was significantly negative: –5.527 (95% CI: −5.854 to −5.199) overall, −4.889 (95% CI: −5.312 to −4.463) in males, and −6.445 (95% CI: −6.808 to −6.081) in females. The local drift, reflecting age-specific annual change, was also negative across all groups: –3.461 (95% CI: −4.149 to −2.769) overall, −1.988 (95% CI: −3.713 to −0.232) in males, and −4.047 (95% CI: −4.563 to −3.527) in females (Figure 2; Supplementary Figures S4, S5).

Age effect: After adjusting for period and cohort effects, COPD mortality increased with age across the 1–95 age groups for the overall population and for males, peaking in the 90–95 age group [overall: 1,511.343 (95% CI: 1,444.325 to 1,581.472); males: 3,161.385 (95% CI: 2,971.217 to 3,363.724)]. In contrast, among females, mortality rates increased continuously with age, indicating a greater risk among older women.

Period effect: After controlling for age and cohort, the period effect demonstrated a steady decline in the relative risk (RR) of COPD mortality over time. Using 2004.5 as the reference (RR = 1.00), all subsequent periods showed reduced RR, reflecting a national trend of decreasing COPD mortality.

Cohort effect: Controlling for age and period, the cohort effect showed that RR of COPD mortality declined among individuals born between 1897 and 2002 in both the general population and among females. Among males, the cohort effect initially increased for those born between 1897 and 1902, peaking at RR = 5.557 (95% CI: 4.828 to 6.397), followed by a downward trend for later cohorts.

### 3.5 Attributable risk factors in China, 1990–2021

**Figure 3:**
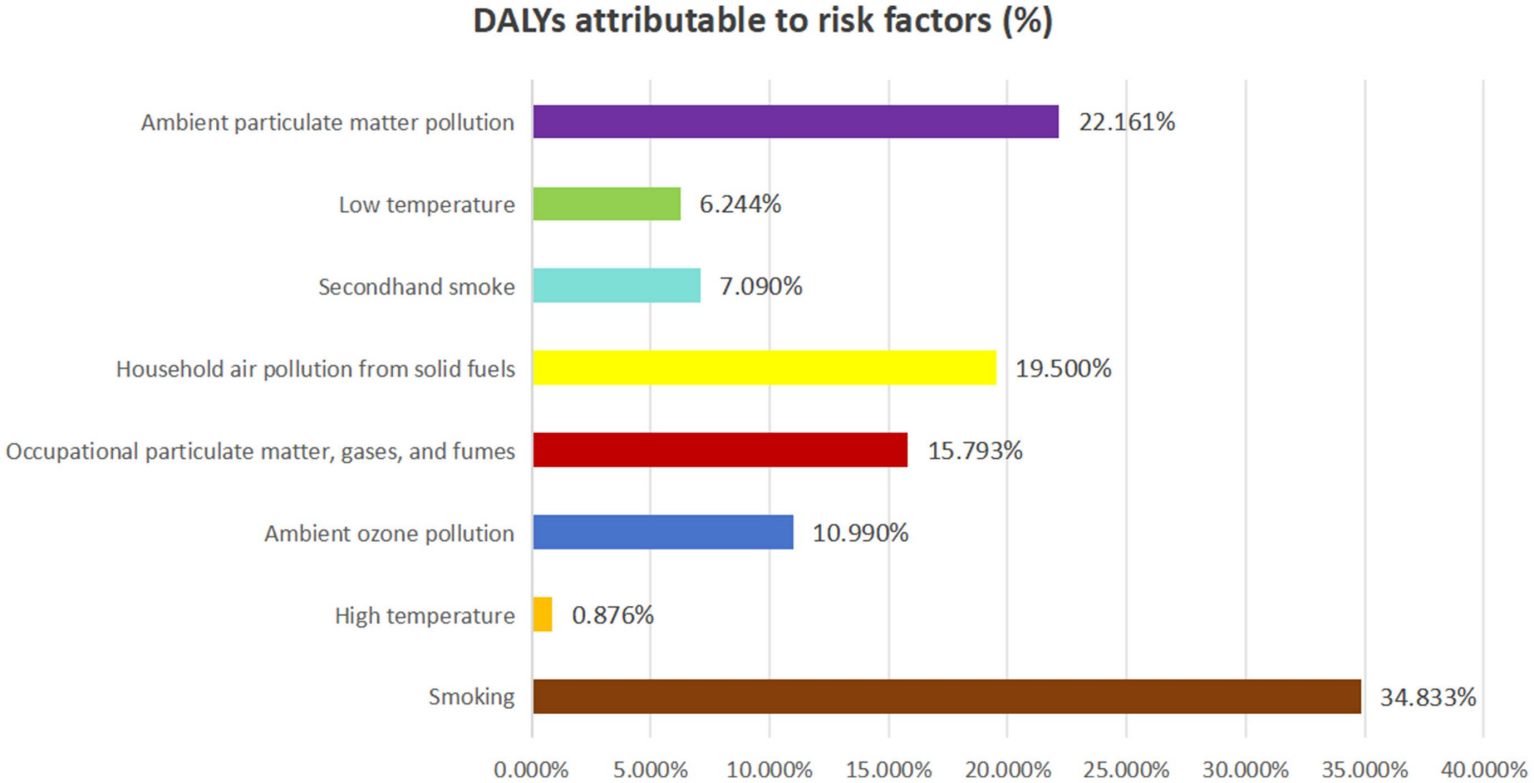
Proportion of disability-adjusted life years (DALYs) attributable to related risk factors for chronic obstructive pulmonary disease in 2021.

The burden of COPD in China, measured in DALYs, was predominantly attributable to modifiable risk factors, with notable variations across age groups. As illustrated in Figure 3, the three leading contributors to COPD-related DALYs nationwide were smoking (34.83%), ambient particulate matter pollution (22.16%), and indoor air pollution from solid fuels (19.50%). The proportion of DALYs attributable to smoking increased with age, peaking in the 70–74 age group, followed by a slight decline in older groups. In contrast, ambient particulate matter pollution peaked in the 85–89 age group, though its contribution remained relatively stable across other ages.

**Figure 4:**
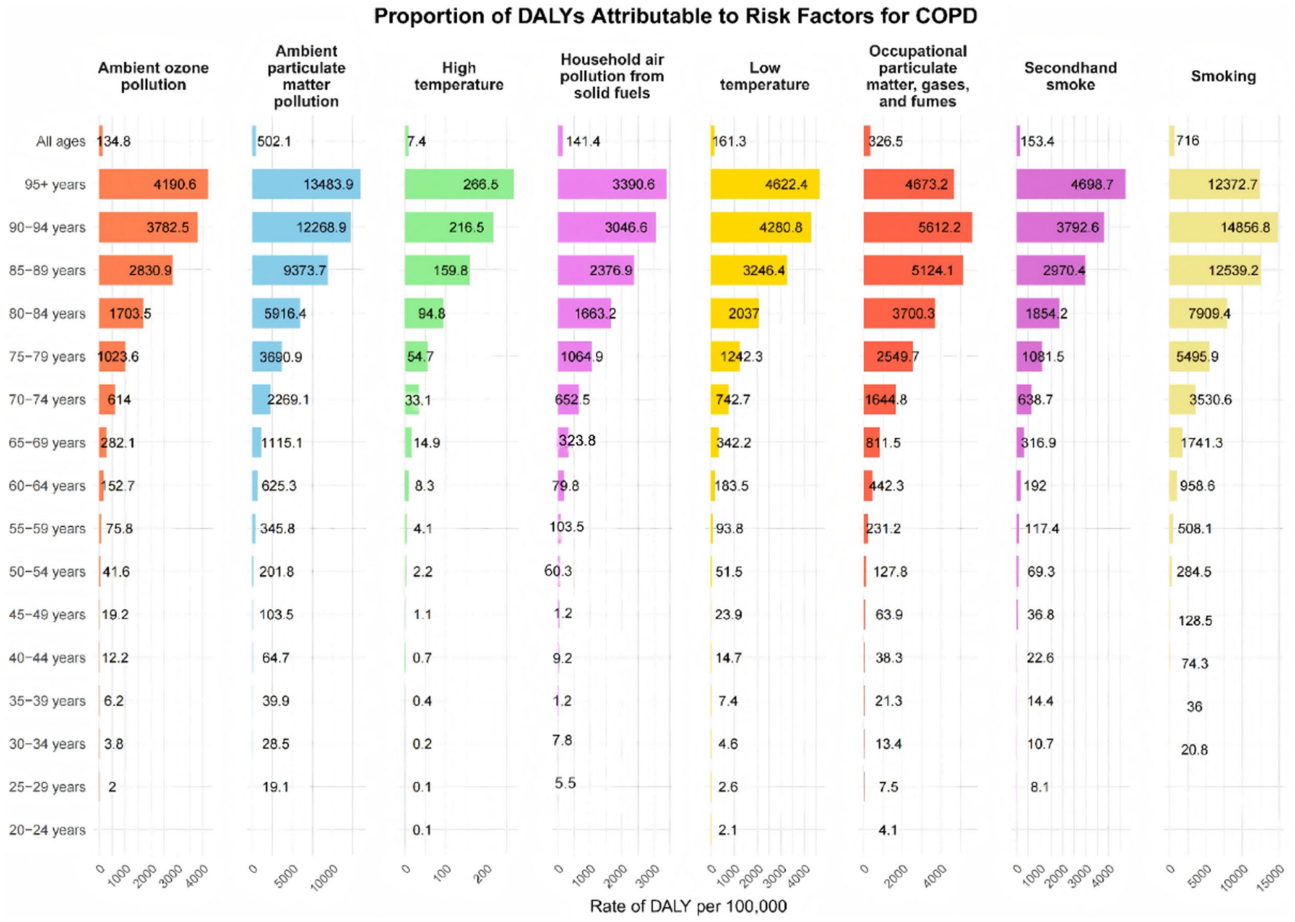
Number of disability cases caused by chronic obstructive pulmonary disease due to risk factors across different age groups in China, 2019.

Occupational exposure to particulates, gases, and fumes demonstrated an age-related pattern, accounting for an increasing share of DALYs with age, reaching a peak of 22.08% in the 65–69 age group (Figure 4; Supplementary Figure S6). We also analyzed mortality attributable to modifiable risk factors across different age groups in China’s COPD burden, which closely mirrored the pattern observed in DALYs (Supplementary Figure S7).

Trends in population-attributable fractions (PAFs) over time revealed a shifting landscape of risk factors. In 1990, the primary contributors to COPD-related DALYs were indoor air pollution from solid fuels, smoking, occupational exposures, ambient particulate matter, low temperatures, secondhand smoke, ambient ozone pollution, and high temperatures. Notably, by 2003, smoking surpassed indoor air pollution as the leading contributor to COPD DALYs. This transition continued, and by 2009, ambient particulate matter pollution became the second leading risk factor, overtaking indoor air pollution (Supplementary Figure S8).

### 3.6 Projected burden of COPD in China, 2022–2050

**Figure 5:**
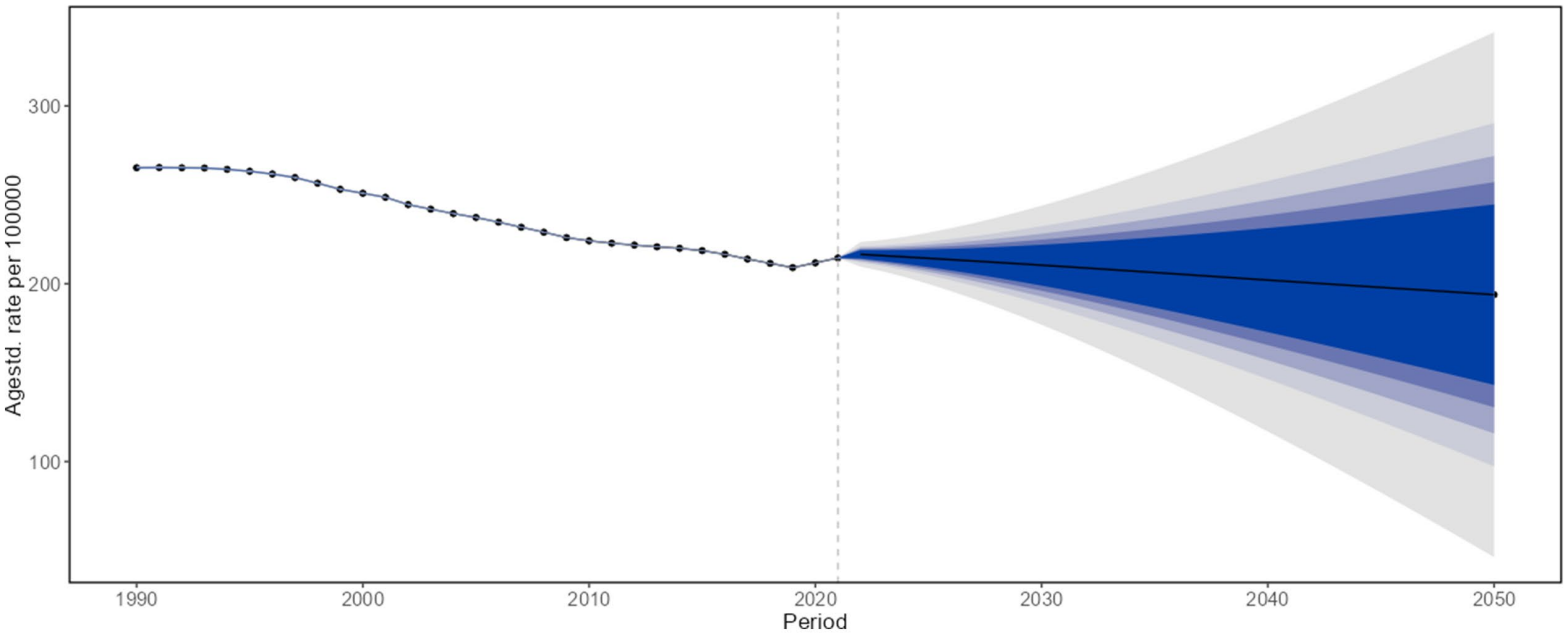
Projected age-standardized incidence rates of chronic obstructive pulmonary disease in the overall population of China, 2022–2050.

According to projections based on the BAPC model, the COPD burden in China is expected to decline steadily from 2022 to 2050. By 2050, the projected ASRs are as follows: ASIR: 193.829 per 100,000 (95% CI: 46.246 to 341.412); ASPR: 2,004.305 per 100,000 (95% CI: 1,871.469 to 2,137.141); ASMR: 50.874 per 100,000 (95% CI: −63.051 to 164.799); and ASDALYs: 887.431 per 100,000 (95% CI: −683.426 to 2,458.288) (Figure 5; Supplementary Figures S9–S11).

## 4 Discussion

### 4.1 Summary of key trends in COPD burden (1990–2021)

This study, based on data from the Global Burden of Disease (GBD) Study 2021, provides a comprehensive and updated assessment of chronic obstructive pulmonary disease (COPD) in China from 1990 to 2021, with projections through 2050. As of 2021, COPD remains the fourth leading cause of death globally and the third in China. Despite significant declines in age-standardized incidence rate (ASIR), prevalence rate (ASPR), mortality rate (ASMR), and disability-adjusted life years (ASDALYs), the absolute number of cases has continued to rise. In 2021 alone, COPD was responsible for approximately 4.43 million new cases, 50.59 million prevalent cases, 1.29 million deaths, and 23.64 million DALYs in China.

### 4.2 Medical advances, contradictions in COPD burden, and their causes

Over the past three decades, China’s rising life expectancy has reflected broader healthcare system improvements (41, 42). Since 1990, particularly in the past two decades, healthcare reforms have expanded access to medical services (43, 44). Interventions targeting chronic and respiratory diseases, including COPD, have contributed to a decline in ASMR (44). However, the overall burden, measured by DALYs, continues to grow. This paradox stems from COPD’s irreversible and progressive nature, compounded by population aging. As more individuals reach older age, the population at risk increases, resulting in more people living with and dying from the disease (45–47).

### 4.3 Dual characteristics of COPD epidemiological trends and the impact of population aging

These findings align with national and global patterns showing a dual trend: declining age-standardized rates and increasing absolute burden (9). Compared to global averages, China continues to experience higher COPD-related mortality and DALYs, likely due to industrialization, urbanization, and sustained environmental and behavioral exposures. This highlights the need to look beyond ASRs and consider the broader public health impact of rising absolute case counts. The concurrent decline in standardized rates and rise in total cases underscores the pivotal role of demographic aging in shaping COPD epidemiology (46, 47). An expanding elderly population increases disease susceptibility and pressures healthcare systems. China must adapt its healthcare strategies and service models to address the growing demand for chronic disease management, particularly for age-related conditions such as chronic obstructive pulmonary disease (COPD). This includes developing sustainable healthcare financing mechanisms within the context of pension and social health insurance reforms aimed at improving the current landscape.

### 4.4 Major modifiable risk factors for COPD and population variations

Tobacco smoking, ambient air pollution, and occupational exposures remain the primary modifiable risk factors for COPD, findings consistent with previous research. Men carry a disproportionate burden, driven by high smoking prevalence. China is home to nearly 300 million smokers and accounts for roughly 40% of global cigarette consumption (48). In contrast, non-smoking women, especially in rural areas, face elevated risk from household biomass fuel use, highlighting the need for clean energy transitions (49).

Tobacco use remains the leading preventable cause of COPD in China (50, 51). Despite public health campaigns, smoking rates remain high, particularly among adolescents and young adults. Early smoking initiation leads to prolonged exposure, compounding disease risk later in life. Secondhand smoke also contributes significantly to morbidity (50). Stronger tobacco control policies, expansion of smoke-free environments, and youth-focused prevention programs are critical. These strategies must address social norms, behavioral drivers, and enforcement barriers.

Occupational exposure ranked fourth among risk factors in 2021. Workers in mining, construction, and heavy industry face harmful exposures to dust, fumes, and particulates, substantially increasing COPD risk (52, 53). Strengthening occupational health requires better enforcement of safety regulations, broader use of personal protective equipment (PPE), and routine health monitoring. Urbanization has introduced additional underrecognized occupational risks, calling for improved surveillance and targeted policy responses.

Ambient air pollution, particularly fine particulate matter (PM2.5), was the second leading contributor to COPD-related DALYs in 2021. Industrial emissions, vehicular exhaust, and coal combustion remain key drivers, especially in urban centers like Beijing and regions such as Sichuan (54). Despite some regulatory progress, greater emission reductions, stricter air quality standards, and investment in green infrastructure are urgently needed. The cumulative impact of air pollution is clearly reflected in long-term disease burden trends. The ≥80-year age group accounted for the highest proportion of PM2.5-attributable COPD deaths, primarily attributed to the cumulative effects of air pollution exposure (55). Our study further demonstrates a significant age-dependent increase in COPD incidence rates, ultimately leading to the greatest disease burden among older adults—particularly those aged ≥80 years. PM2.5 and PM10 particles penetrate deep into the lungs, triggering inflammation and accelerating respiratory decline (56). Regional variation in pollution sources and exposure levels demands locally tailored interventions and sustained policy enforcement. Urban–rural disparities further shape COPD risk: while many urban households have transitioned to cleaner energy, rural populations still rely heavily on coal and biomass, sustaining high indoor air pollution levels (57, 58). Addressing these inequities requires investment in clean energy technologies, rural health infrastructure, and community education. Chronic obstructive pulmonary disease (COPD) control constitutes a systematic public health imperative requiring robust interagency coordination. Effective management necessitates synergistic interventions across sectors, as evidenced by environmental governance implementing region-specific PM₂.₅ reduction targets through industrial emission controls and vehicle electrification policies; energy infrastructure subsidizing clean fuel adoption in rural households to displace solid fuel combustion; and labor regulations enforcing occupational safety standards in high-risk industries (e.g., mining, construction) through particulate monitoring and respiratory protection mandates. This multi-sectoral approach demonstrates how coordinated action across environmental, energy, and labor domains can address COPD’s complex etiology (56, 59).

### 4.5 Association between population aging and COPD risk, and intervention directions

From 2010 to 2020, the proportion of individuals aged ≥60 in China rose from 13.3 to 18.7%, and those aged ≥65 increased from 8.9 to 13.5% (46). By 2050, more than 400 million people in China are projected to be aged ≥65, including over 150 million aged ≥80 (47). Aging is closely linked to COPD via physiological changes such as reduced lung elasticity, chronic inflammation, and impaired tissue repair (60). Combined with cumulative environmental exposures, these changes elevate COPD risk among older adults. As life expectancy increases, more individuals survive to ages where COPD manifests clinically. This demographic shift is reshaping disease epidemiology and calls for age-specific interventions, including geriatric respiratory care, community-based screening, and integrated chronic disease management at the primary care level.

### 4.6 Cumulative impact of air pollution and challenges in health service equity

Air pollution continues to drive COPD progression, particularly in industrial and coal-reliant regions (21, 61). Age–period–cohort analyses show declining mortality risk for most birth cohorts since 1897, but gains remain uneven. Vulnerable and rural populations often do not benefit equally from public health improvements. Rapid urbanization has produced mixed outcomes: urban areas enjoy better healthcare and energy access, while rural regions face persistent exposure and limited services (62). Geographic, economic, and educational disparities hinder equitable access to care and contribute to disease underdiagnosis. Addressing these challenges requires inclusive policy design and sustained investment in health systems.

### 4.7 Policy Progress, diagnostic barriers, and service inequalities

The inclusion of COPD in China’s Basic Public Health Service Program (2024) marks a critical policy milestone. Integrating COPD care with hypertension and diabetes management prioritizes early screening, smoking cessation, public education, and pulmonary rehabilitation. Standardized care pathways and reimbursement policies may enhance early diagnosis and reduce exacerbations. However, diagnostic barriers persist, especially in rural areas. Pulmonary function testing serves as the gold standard for COPD diagnosis. Nevertheless, due to the high technical demands of the procedure, expensive equipment costs, limited awareness among primary care clinicians, and stringent requirements for patient-operator coordination, the accessibility of pulmonary function testing remains insufficient at grassroots hospitals and township health centers. According to the China Pulmonary Health (CPH) study, only 12.0% of surveyed residents had undergone pulmonary ventilation function tests (21). In addition, regional disparities in data availability hamper local trend identification (63, 64). When primary care hospitals or township health centers lack the capacity for pulmonary function testing, COPD screening questionnaires or clinical decision support system-based tools can be employed to screen for COPD. Suspected COPD patients should be promptly referred to higher-level hospitals for definitive diagnosis. Commonly used questionnaires include the COPD Screening Questionnaire (COPD-SQ) (65), the COPD Diagnostic Questionnaire (CDQ) (66), the Lung Function Questionnaire (LFQ) (67), and the COPD Population Screener Questionnaire (COPD-PS) (68).

### 4.8 Strengths, limitations, and future research directions of this study

This study demonstrates methodological rigor through several principal strengths: the utilization of comprehensive, high-quality GBD 2021 data enables robust analysis of national-level trends spanning 1990–2021, while the incorporation of Bayesian modeling provides future projections for strategic health planning. Our multidimensional assessment quantifies disease burden through complementary metrics and rigorous attributable risk factor analysis, yielding policy-relevant insights for aging populations and high-risk subgroups. The analytical robustness is further evidenced by the consistent application of Estimated Annual Percentage Change (EAPC) methodology throughout. This study has several inherent limitations. The reliance on modeled GBD data may inadequately capture subclinical manifestations and undiagnosed COPD cases. Furthermore, the absence of province-level data constrains granular geographical analysis, while emerging risk factors—including genetic susceptibility, indoor occupational exposures, and climate-related influences—require further investigation. Methodologically, we identified key constraints: (1) heterogeneity in data quality across sources; (2) underrepresentation of rural and marginalized populations; (3) lack of clinical subtype or severity stratification; (4) absence of individual-level behavioral data; (5) inherent uncertainties in long-term projections; (6) temporal dissociation between exposures and disease manifestation; and (7) inability to account for comorbidities and competing risks. Future research should focus on large-scale spirometry-based surveys, provincial-level data analysis, and cohort studies stratified by urbanization, occupation, and exposure patterns. A multi-sectoral strategy is essential to reduce the COPD burden. Coordinated action across public health, environmental regulation, labor policy, and rural development is needed. Special attention should be given to high-risk groups such as rural women, older men, and industrial workers. Only through sustained, collaborative, and targeted policy action can COPD be effectively addressed within China’s shifting demographic and environmental landscape.

## 5 Conclusion

This study demonstrates that, despite substantial declines in age-standardized COPD rates in China since 1990 (ASMR: -68.4%; ASDALYs: −68.1%), the absolute disease burden has increased by 104% as a result of rapid population aging—a paradox epitomized by individuals >80 years bearing 32.7% of PM2.5-attributable deaths. Leveraging a novel application of Bayesian age-period-cohort modeling to GBD 2021 data, we identify three key insights insights: (1) ambient PM₂.₅ has overtaken household pollution as the secondary risk driver (22.2% of DALYs), while occupational exposures unexpectedly peak in the 65–69 age group (22.1% DALYs); (2) the fraction of COPD burden attributable to tobacco use has declined from 42.1% in 1990 to 34.8% in 2021, reflecting a shifting risk landscape; and (3) projections reveal persistent regional disparities, with the 2050 ASIR projected to stagnate at 193.8 per 100,000 (95% CI: 46.2–341.4) in underserved provinces. These findings broaden the global understanding of COPD governance by illustrating how low-and middle-income countries undergoing demographic transition face a “triple-disease shift”—from infectious, to pollution-driven,to aging-exacerbated epidemics. Importantly, this work provides an epidemiological foundation for multisectoral action: environmental regulations targeting industrial PM₂.₅ emissions could avert an estimated 214,000 deaths annually by 2040; rural clean energy subsidies demonstrate a 3.2-fold greater cost-effectiveness compared with other intervention; and occupational safety reforms in mining and construction could reduce DALY in high-risk cohorts by 22%. By quantifying the intersection of aging, environmental degradation, and health system fragmentation, this study underscores and urgent need for integrated, cross-sector respiratory health policies in China.

## Data Availability

The datasets presented in this study can be found in online repositories. The names of the repository/repositories and accession number(s) can be found at: https://vizhub.healthdata.org/gbd-results/.
