# Supplementary material for: Burden of chronic obstructive pulmonary disease and its attributable risk factors in China from 1990 to 2021, with projections to 2050: an analysis of data from the Global Burden of Disease study 2021
Source: Front Med (Lausanne). 2025 Sep 3;12:1644022. doi: 10.3389/fmed.2025.1644022 (PMC12440722; doi:10.3389/fmed.2025.1644022)
Supplement: Supplementary file 1 [file Table_1.DOCX]

Supplementary Material

Table S1 | Prevalence Cases and Rates of Chronic Obstructive Pulmonary Disease (COPD) in 1990 and 2021, and the Time Trend from 1990 to 2021.

| Prevalence(95% UI) | | | | | |
| --- | --- | --- | --- | --- | --- |
|  | 1990 | | 2021 | | EAPC，95%CI  （1990-2021） |
|  | Number | rate per 100 000 | Number | rate per 100 000 |  |
| All age | 23144968.765(20878168.631,25434630.636) | 1967.344(1774.664,2161.968) | 50588429.297(44975892.806,57116835.167) | 3555.690(3161.203,4014.549) | 1.935(1.911,1.960) |
| age-standardized |  | 2761.807(2498.943,3033.596) |  | 2499.347(2236.212,2793.287) | -0.333(-0.375,-0.291) |
| 15-19 years | 394773.268(339552.446,431255.724) | 311.667(268.071,340.469) | 195234.256(154867.366,230780.248) | 261.455(207.396,309.058) | -0.630(-0.686,-0.573) |
| 20-24 years | 620444.631(533224.395,695288.282) | 470.031(403.955,526.730) | 276167.765(221669.645,333050.612) | 377.412(302.934,455.148) | -0.841(-0.916,-0.766) |
| 25-29 years | 710538.434(606886.556,808726.944) | 646.594(552.270,735.946) | 446656.710(361901.903,540443.611) | 516.473(418.470,624.920) | -0.899(-0.994,-0.803) |
| 30-34 years | 729942.117(636549.691,828163.709) | 827.183(721.349,938.490) | 811579.205(674100.569,976928.598) | 669.878(556.403,806.358) | -0.834(-0.937,-0.731) |
| 35-39 years | 927622.773(810027.297,1042572.629) | 1015.582(886.836,1141.431) | 891647.424(755188.864,1062627.407) | 841.469(712.689,1002.826) | -0.710(-0.797,-0.623) |
| 40-44 years | 909776.168(806325.637,1000089.901) | 1355.966(1201.779,1490.573) | 1019720.325(879296.775,1169647.669) | 1114.038(960.626,1277.833) | -0.722(-0.795,-0.649) |
| 45-49 years | 1221374.386(1003704.645,1448283.690) | 2366.135(1944.450,2805.721) | 1983199.419(1659181.417,2338079.787) | 1797.654(1503.950,2119.332) | -1.029(-1.108,-0.950) |
| 50-54 years | 1871987.255(1530921.715,2202237.631) | 3923.607(3208.748,4615.798) | 3573095.739(2907177.392,4306534.406) | 2956.416(2405.428,3563.271) | -1.073(-1.151,-0.996) |
| 55-59 years | 2416507.891(2043514.239,2769083.258) | 5571.957(4711.913,6384.922) | 4901110.867(3993197.087,5814515.368) | 4457.891(3632.082,5288.694) | -0.837(-0.916,-0.758) |
| 60-64 years | 2714503.963(2350676.124,3078484.820) | 7681.661(6652.080,8711.675) | 4684148.213(3978071.704,5383776.254) | 6416.187(5449.028,7374.514) | -0.617(-0.689,-0.545) |
| 65-69 years | 3207815.514(2725614.655,3689394.098) | 11758.074(9990.593,13523.274) | 7534896.768(6320065.603,8908847.846) | 9823.405(8239.604,11614.654) | -0.587(-0.648,-0.525) |
| 70-74 years | 3129105.997(2648028.728,3544228.965) | 16628.569(14072.047,18834.598) | 7839483.199(6520704.354,9236310.288) | 14709.205(12234.784,17330.069) | -0.363(-0.416,-0.310) |
| 75-79 years | 2430855.196(2043733.838,2764519.568) | 21359.458(17957.897,24291.303) | 6833131.601(5589021.394,7984413.275) | 20632.098(16875.606,24108.302) | -0.068(-0.114,-0.023) |
| 80-84 years | 1312912.838(1087701.644,1499246.043) | 24785.383(20533.809,28303.012) | 5247154.469(4378982.355,5971195.595) | 26511.685(22125.173,30169.963) | 0.301(0.252,0.349) |
| 85-89 years | 456308.092(372674.281,534317.300) | 27050.810(22092.839,31675.344) | 3051322.890(2556353.158,3477825.124) | 32032.360(26836.237,36509.721) | 0.676(0.620,0.733) |
| 90-94 years | 80264.368(62328.665,99533.982) | 26159.907(20314.270,32440.294) | 1047868.103(855639.346,1211007.495) | 35739.174(29182.913,41303.297) | 1.191(1.123,1.260) |
| 95+ years | 10235.876(7303.763,13628.816) | 25278.708(18037.507,33657.974) | 252012.347(200880.366,307363.823) | 39432.477(31431.834,48093.345) | 1.615(1.552,1.678) |
| **sex** | Number | ASR* per 100 000 | Number | ASR* per 100 000 | EAPC |
| Female | 12255303.936(11105058.300,13454860.887) | 2854.448(2596.708,3135.840) | 26362819.992(23305958.836,29845647.074) | 2492.114(2215.969,2798.031) | -0.349(-0.443,-0.256) |
| Male | 10889664.828(9732816.283,11966510.366) | 2638.554(2371.952,2902.846) | 24225609.305(21588802.286,27240289.986) | 2479.432(2214.909,2762.134) | -0.335(-0.497,-0.173) |

Note: *ASR, age-standardized rates;EAPC, estimated annual percentage change.

Table S2 | Mortality Cases and Rates of Chronic Obstructive Pulmonary Disease (COPD) in 1990 and 2021, and the Time Trend from 1990 to 2021.

| Deaths (95% UI) | | | | | |
| --- | --- | --- | --- | --- | --- |
|  | 1990 | | 2021 | | EAPC，95%CI  （1990-2021） |
|  | Number | rate per 100 000 | Number | rate per 100 000 |  |
| All age | 1237950.412(1065524.892,1384796.647) | 105.227(90.571,117.709) | 1285433.174(1044727.799,1539819.909) | 90.349(73.430,108.229) | -1.118(-1.333.-0.902) |
| age-standardized |  | 231.776(198.978,257.418) |  | 73.231(59.732,86.852) | -4.250(-4.483,-4.017) |
| 15-19 years | 1167.361(972.620,1368.412) | 0.922(0.768,1.080) | 101.294(78.980,131.534) | 0.136(0.106,0.176) | -6.725(-7.032,-6.417) |
| 20-24 years | 1572.311(1312.428,1830.277) | 1.191(0.994,1.387) | 199.202(154.256,262.992) | 0.272(0.211,0.359) | -5.676(-6.124,-5.225) |
| 25-29 years | 1619.667(1344.156,1881.835) | 1.474(1.223,1.712) | 308.691(244.003,387.792) | 0.357(0.282,0.448) | -5.446(-5.845,-5.046) |
| 30-34 years | 2695.621(2249.624,3114.793) | 3.055(2.549,3.530) | 837.084(669.857,1068.748) | 0.691(0.553,0.882) | -5.567(-5.926,-5.206) |
| 35-39 years | 5396.954(4482.704,6278.318) | 5.909(4.908,6.874) | 1295.483(1007.837,1689.395) | 1.223(0.951,1.594) | -5.861(-6.223,-5.496) |
| 40-44 years | 9133.557(7546.501,10717.171) | 13.613(11.248,15.973) | 2470.561(1939.031,3175.783) | 2.699(2.118,3.470) | -5.722(-6.005,-5.438) |
| 45-49 years | 12997.710(10674.965,15346.783) | 25.180(20.680,29.731) | 5309.177(4070.131,6816.736) | 4.812(3.689,6.179) | -5.698(-5.964,-5.431) |
| 50-54 years | 31565.622(25870.610,36785.832) | 66.160(54.224,77.102) | 13927.539(10640.440,17722.078) | 11.524(8.804,14.663) | -6.450(-6.815,-6.083) |
| 55-59 years | 56147.476(46558.509,66538.924) | 129.464(107.354,153.425) | 26232.162(20134.347,33690.788) | 23.860(18.314,30.644) | -6.275(-6.584,-5.965) |
| 60-64 years | 97875.195(82782.731,112209.951) | 276.973(234.263,317.538) | 39937.311(30659.045,49745.893) | 54.705(41.996,68.140) | -5.714(-5.920,-5.507) |
| 65-69 years | 147482.089(125441.096,167462.163) | 540.588(459.798,613.823) | 92367.934(72991.815,115150.833) | 120.422(95.161,150.125) | -5.382(-5.615,-5.148) |
| 70-74 years | 235789.677(201361.124,266753.111) | 1253.024(1070.065,1417.569) | 169326.283(135940.360,208895.757) | 317.707(255.065,391.951) | -4.968(-5.196,-4.739) |
| 75-79 years | 252629.956(216961.641,281332.631) | 2219.811(1906.400,2472.016) | 220456.358(179169.814,266851.564) | 665.650(540.989,805.737) | -4.422(-4.634,-4.209) |
| 80-84 years | 211822.619(177609.417,234515.477) | 3998.822(3352.940,4427.221) | 279522.797(225353.536,327443.450) | 1412.312(1138.618,1654.435) | -3.893(-4.108,-3.678) |
| 85-89 years | 127898.064(108344.378,142408.161) | 7582.040(6422.860,8442.226) | 272674.613(222238.301,318719.066) | 2862.500(2333.027,3345.868) | -3.704(-4.037,-3.369) |
| 90-94 years | 36889.001(30583.609,41522.496) | 12022.929(9967.865,13533.086) | 127869.949(101335.602,151212.099) | 4361.204(3456.209,5157.324) | -3.742(-4.007,-3.477) |
| 95+ years | 5267.532(3927.219,5989.957) | 13008.793(9698.731,14792.907) | 32596.738(23727.720,40764.340) | 5100.425(3712.686,6378.413) | -3.479(-3.679,-3.279) |
| **sex** | Number | ASR* per 100 000 | Number | ASR* per 100 000 | EAPC |
| Female | 596733.964(448051.822,702428.233) | 199.336(150.901,233.384) | 533406.238(391941.014,693211.255) | 52.726(38.489,68.223) | -5.002(-5.284,-4.719) |
| Male | 641216.447(545679.528,755295.017) | 284.574(251.089,326.417) | 752026.936(583837.632,917240.810) | 105.732(83.962,126.668) | -3.571(-3.794,-3.347) |

Note: *ASR, age-standardized rates;EAPC, estimated annual percentage change.

Table S3 | Disability Cases and Rates of Chronic Obstructive Pulmonary Disease (COPD) in 1990 and 2021, and the Time Trend from 1990 to 2021.

| DALYs (95% UI) | | | | | |
| --- | --- | --- | --- | --- | --- |
|  | 1990 | | 2021 | | EAPC，95%CI  （1990-2021） |
|  | Number | rate per 100 000 | Number | rate per 100 000 |  |
| All age | 26097667.671(22790559.425,29216352.031) | 2218.326(1937.219,2483.418) | 23640320.961(19998658.356,27921931.160) | 1661.598(1405.638,1962.538) | -1.489(-1.682,-1.296) |
| age-standardized |  | 3852.568(3349.973,4279.010) |  | 1227.659(1048.450,1442.541) | -4.186(-4.382,-3.990) |
| 15-19 years | 117170.024(100435.706,134161.123) | 92.504(79.292,105.918) | 23465.524(19024.653,27809.621) | 31.425(25.478,37.242) | -3.765(-3.989,-3.541) |
| 20-24 years | 157336.589(136104.219,181021.131) | 119.194(103.109,137.136) | 36188.626(29324.031,43213.937) | 49.455(40.074,59.056) | -3.295(-3.526,-3.064) |
| 25-29 years | 159549.404(138301.633,182911.195) | 145.191(125.855,166.450) | 55701.164(45778.358,65703.619) | 64.408(52.934,75.974) | -3.080(-3.274,-2.885) |
| 30-34 years | 213682.175(183298.390,242625.075) | 242.148(207.717,274.947) | 114232.195(95787.338,133761.255) | 94.287(79.063,110.407) | -3.504(-3.712,-3.296) |
| 35-39 years | 359232.174(305979.182,410222.889) | 393.295(334.993,449.121) | 140610.683(117059.074,166630.912) | 132.698(110.471,157.253) | -3.998(-4.233,-3.762) |
| 40-44 years | 509696.626(432693.832,588615.141) | 759.672(644.904,877.295) | 199816.715(169068.941,233944.089) | 218.299(184.707,255.583) | -4.386(-4.616,-4.155) |
| 45-49 years | 654646.803(552296.793,762096.520) | 1268.229(1069.950,1476.389) | 384960.290(321211.408,461035.441) | 348.944(291.159,417.901) | -4.449(-4.672,-4.225) |
| 50-54 years | 1350790.802(1132219.429,1554023.344) | 2831.201(2373.085,3257.168) | 812245.377(673131.197,979190.521) | 672.060(556.956,810.192) | -5.288(-5.583,-4.992) |
| 55-59 years | 2070667.405(1741518.077,2403184.680) | 4774.522(4015.573,5541.237) | 1263788.429(1024253.874,1535736.453) | 1149.501(931.628,1396.856) | -5.277(-5.534,-5.020) |
| 60-64 years | 3038461.233(2604820.610,3470270.418) | 8598.414(7371.273,9820.373) | 1511649.455(1234652.302,1797140.442) | 2070.606(1691.185,2461.662) | -5.024(-5.190,-4.857) |
| 65-69 years | 3832244.922(3305452.267,4302442.487) | 14046.886(12115.956,15770.370) | 2817958.603(2334610.133,3379482.271) | 3673.833(3043.681,4405.903) | -4.802(-4.996,-4.608) |
| 70-74 years | 4954229.244(4266084.252,5557102.292) | 26327.565(22670.653,29531.328) | 3976193.401(3291563.930,4791778.461) | 7460.523(6175.954,8990.804) | -4.569(-4.774,-4.364) |
| 75-79 years | 4239247.707(3669477.939,4698859.041) | 37249.456(32242.998,41287.972) | 4030286.016(3390893.152,4794419.253) | 12169.129(10238.533,14476.368) | -4.112(-4.307,-3.917) |
| 80-84 years | 2758574.031(2336889.293,3040138.195) | 52076.811(44116.177,57392.225) | 3873601.499(3199744.549,4478223.547) | 19571.694(16166.976,22626.597) | -3.658(-3.853,-3.463) |
| 85-89 years | 1312275.994(1120026.586,1451099.410) | 77794.212(66397.302,86023.928) | 2929872.895(2438207.519,3378227.204) | 30757.395(25595.961,35464.156) | -3.522(-3.832,-3.211) |
| 90-94 years | 325503.716(270872.878,365713.596) | 106088.755(88283.374,119194.031) | 1184262.690(954081.397,1387658.134) | 40391.124(32540.432,47328.242) | -3.568(-3.816,-3.319) |
| 95+ years | 44358.822(33306.552,50442.933) | 109549.363(82254.473,124574.795) | 285487.398(213032.415,352858.464) | 44670.332(33333.270,55211.910) | -3.332(-3.522,-3.142) |
| **sex** | Number | ASR* per 100 000 | Number | ASR* per 100 000 | EAPC |
| Female | 12203211.190(9285514.196,14381014.677) | 3358.074(2606.175,3917.478) | 10159577.964(7922016.461,12776802.527) | 963.616(755.758,1209.579) | -4.671(-4.909,-4.433) |
| Male | 13894456.481(11788416.514,16460928.508) | 4551.322(3945.855,5280.403) | 13480742.997(10668238.346,16357128.406) | 1603.157(1281.262,1923.147) | -3.737(-3.919,-3.556) |

Note: *ASR, age-standardized rates;EAPC, estimated annual percentage change.


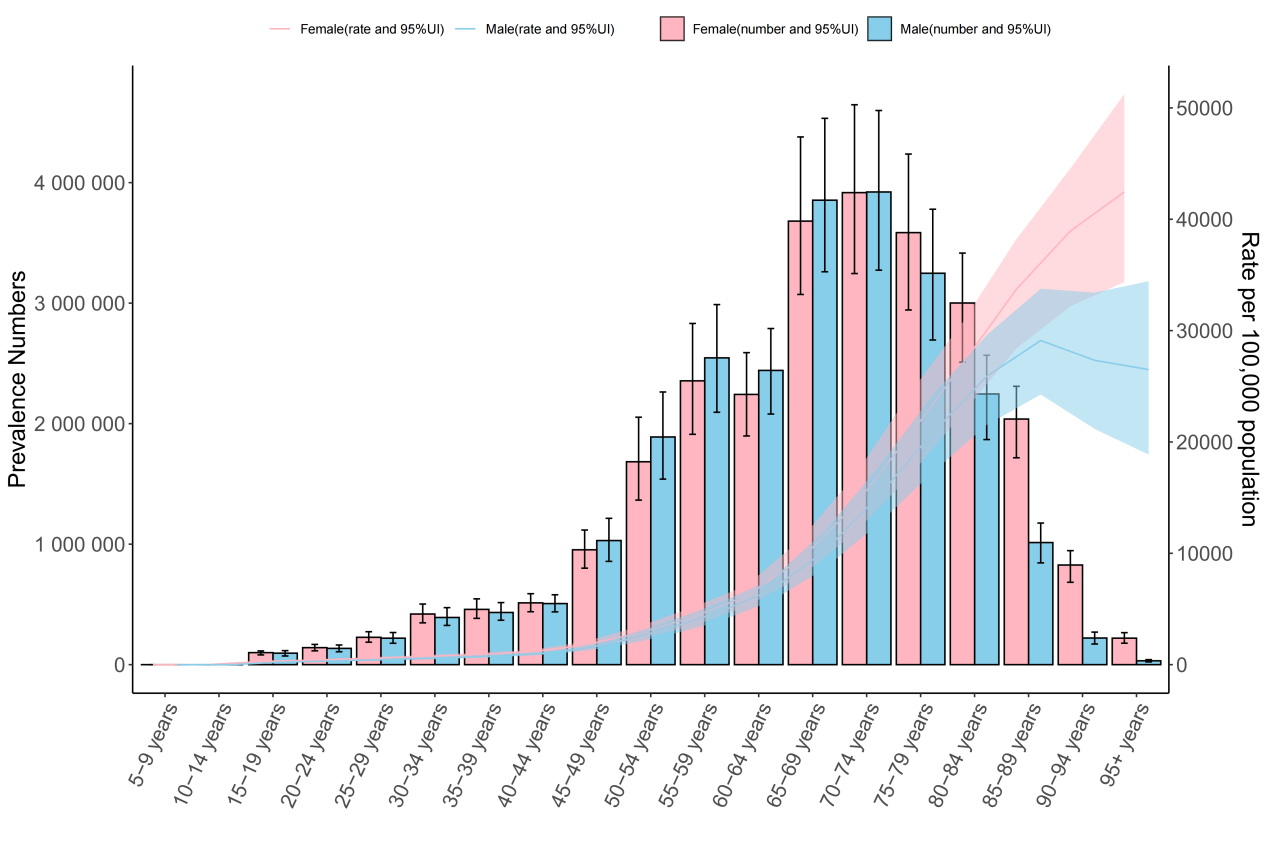


Figure S1: Global Number of Prevalent Cases and Prevalence Rate per 100,000 Population of Chronic Obstructive Pulmonary Disease by Age and Sex in 2021.Note: The lines represent the number of prevalent cases for males and females, with a 95% uncertainty interval.


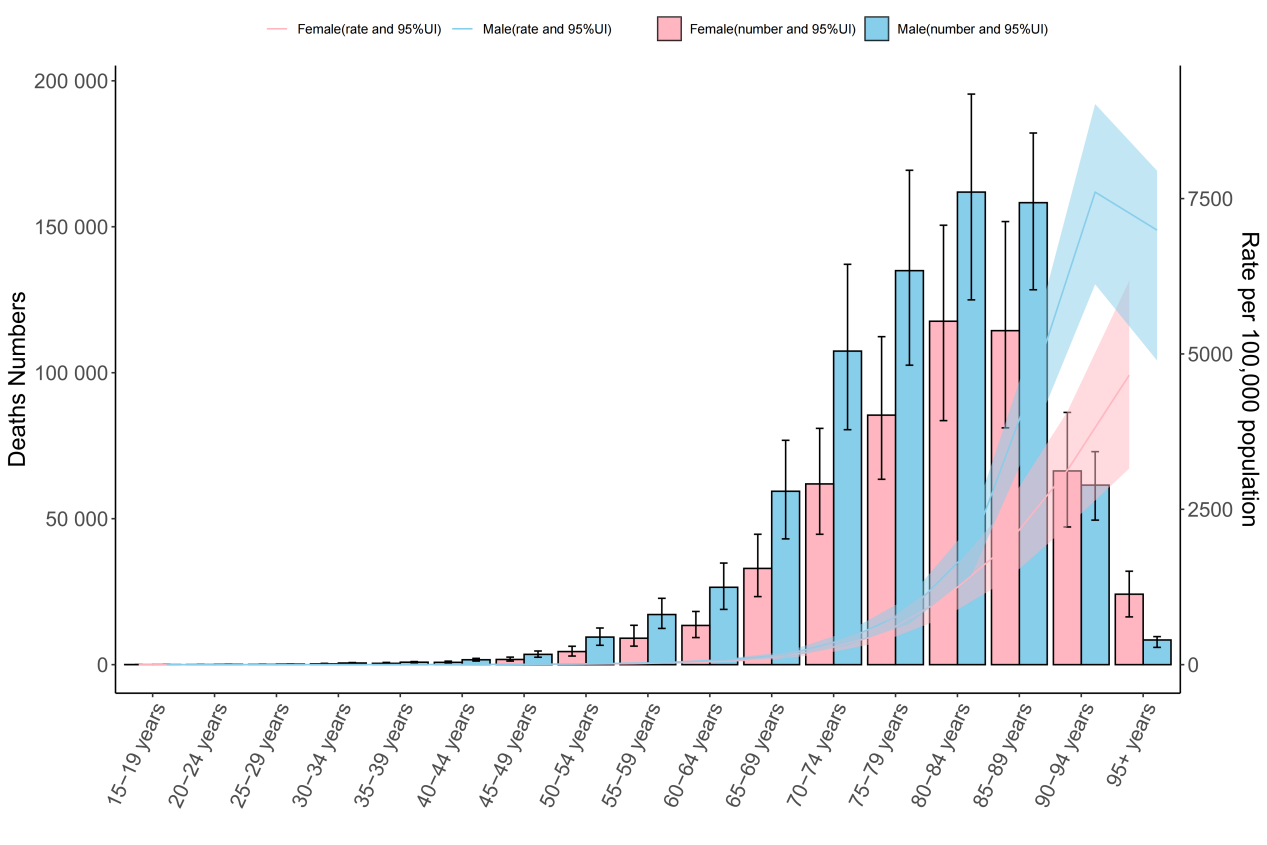


Figure S2: Global Number of Deaths and Mortality Rate per 100,000 Population of Chronic Obstructive Pulmonary Disease by Age and Sex in 2021.

Note: The lines represent the number of deaths for males and females, with a 95% uncertainty interval.


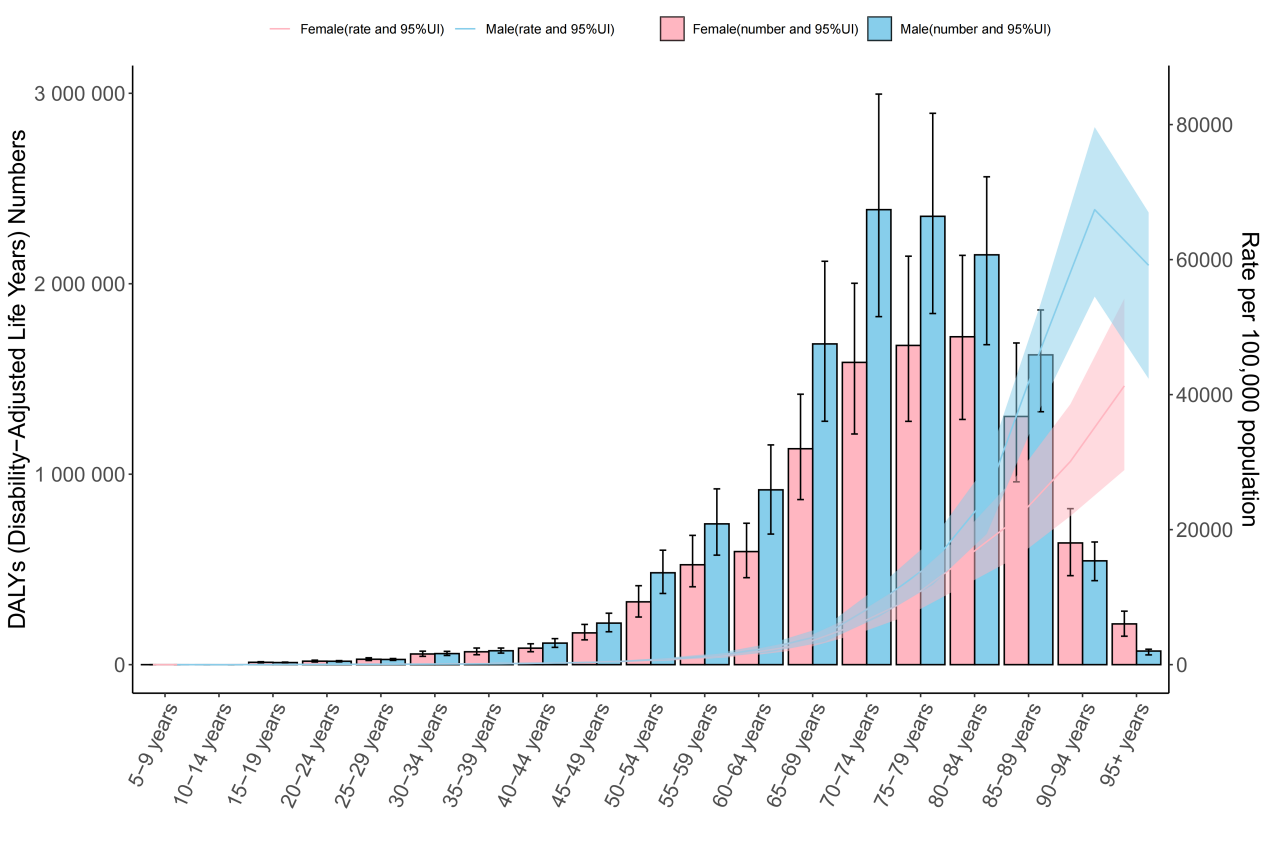


Figure S3: Global Number of Disability Cases and DALYs Rate per 100,000 Population of Chronic Obstructive Pulmonary Disease by Age and Sex in 2021.

Note: The lines represent the number of disability cases for males and females, with a 95% uncertainty interval.


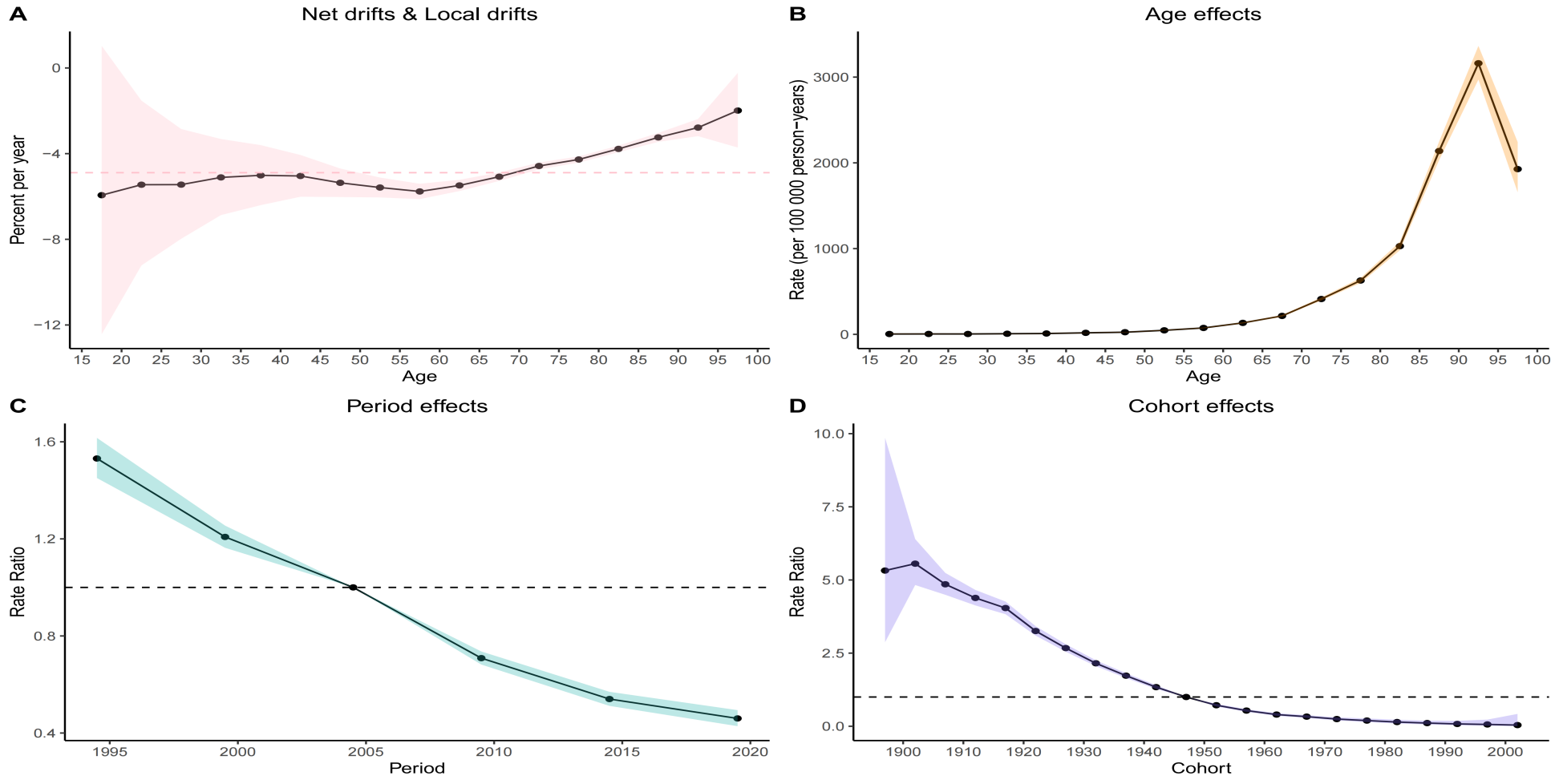


Figure S4: Visualization of the Bayesian Age-Period-Cohort Analysis Model for COPD Mortality Among Chinese Males from 1990 to 2021.

Note: Panel A: Net drifts and Local drifts on on mortality relative risk. Panel B: Age effects on mortality relative risk;Panel C: Period effects on mortality relative risk; Panel D: Cohort effects on mortality relative risk.


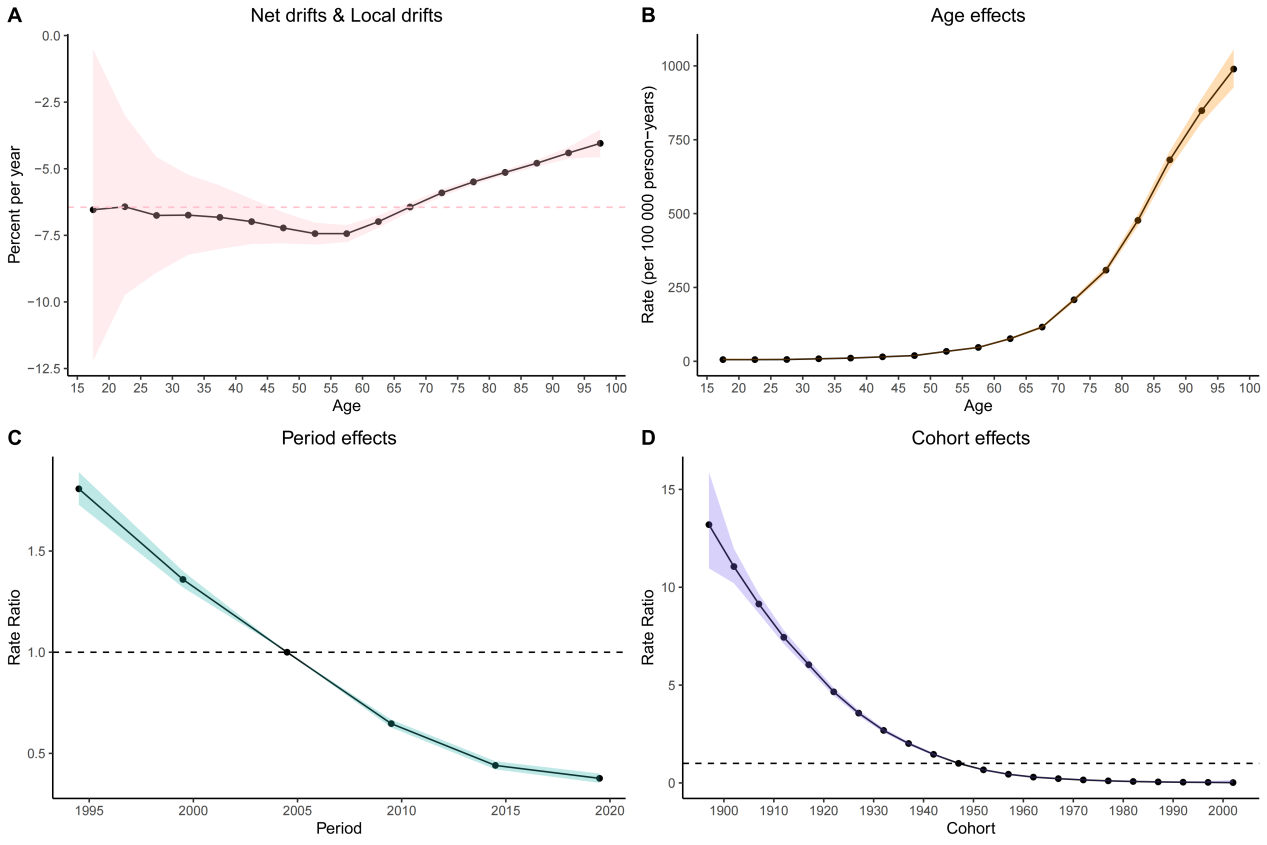


Figure S5: Visualization of the Bayesian Age-Period-Cohort Analysis Model for COPD Mortality Among Chinese Females from 1990 to 2021.

Note: Panel A: Net drifts and Local drifts on on mortality relative risk. Panel B: Age effects on mortality relative risk;Panel C: Period effects on mortality relative risk; Panel D: Cohort effects on mortality relative risk.


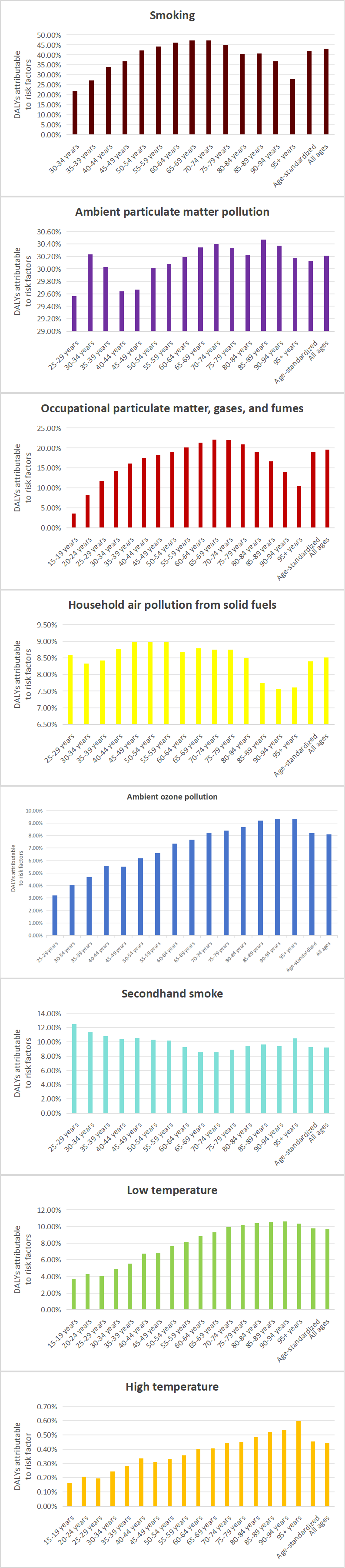


Figure S6: Percentage of Disability-Adjusted Life Years (DALYs) Attributable to Chronic Obstructive Pulmonary Disease by Age Group in China, 2021.


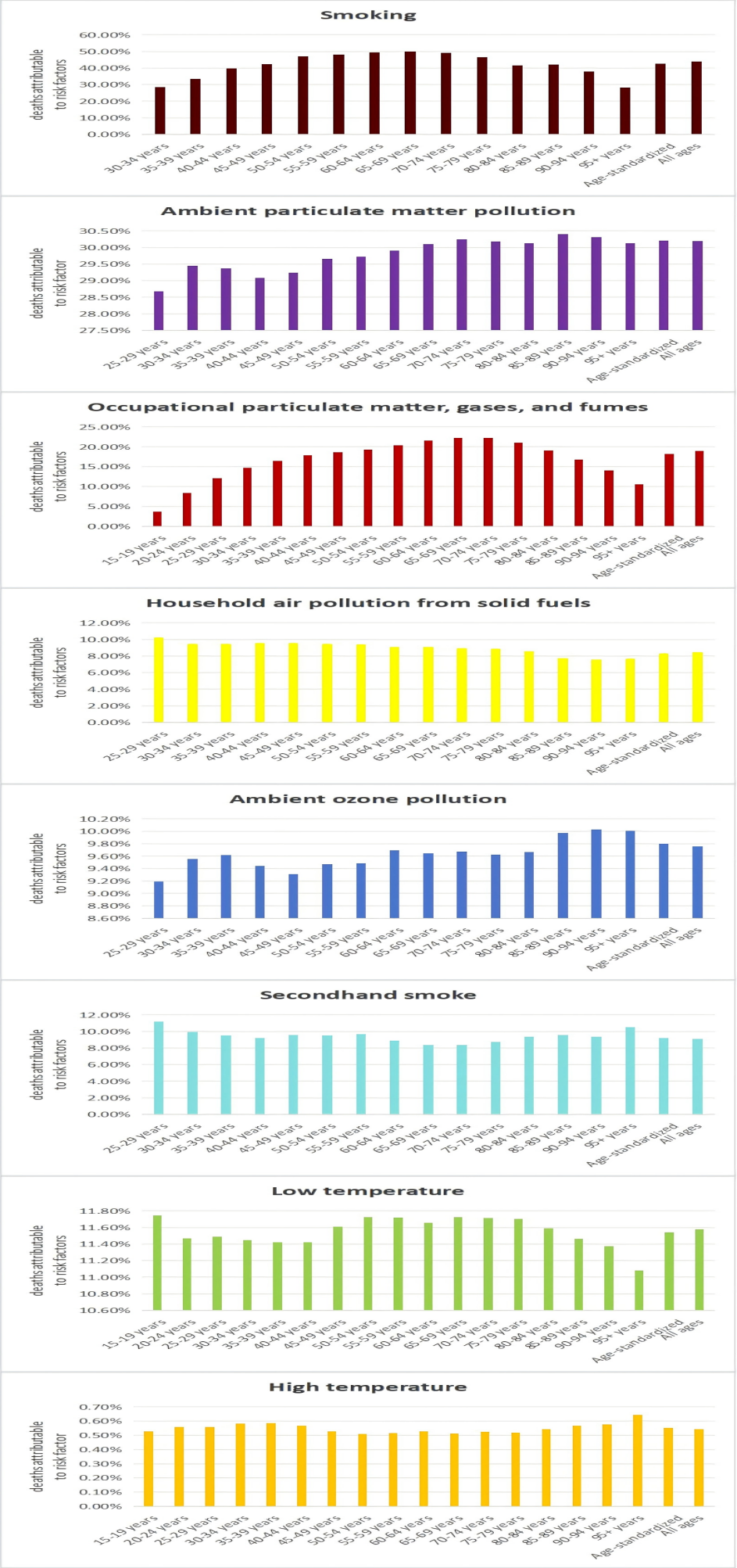


Figure S7: Percentage of Deaths Attributable to Chronic Obstructive Pulmonary Disease by Age Group in China, 2021.


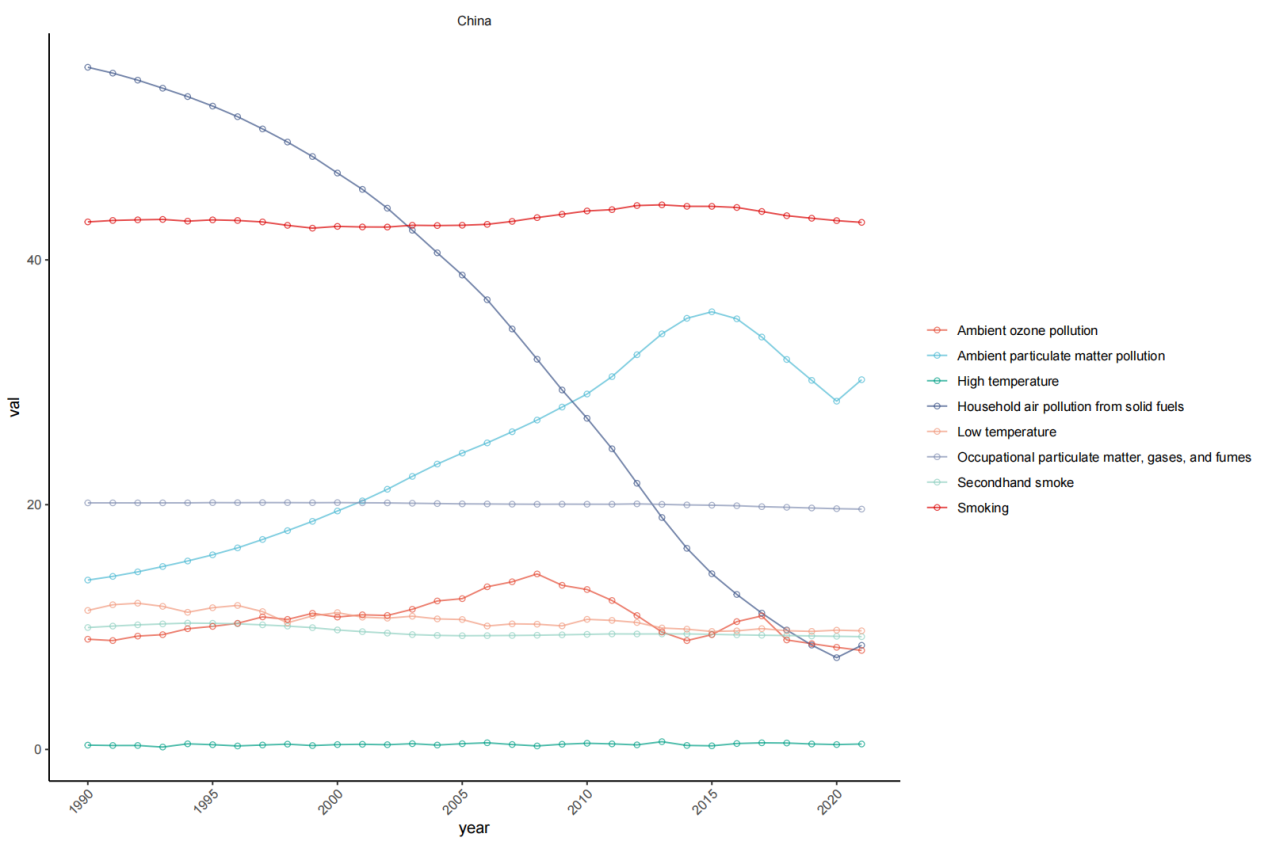


Figure S8: Changes in the Impact of Risk Factors on DALYs for Chronic Obstructive Pulmonary Disease in China, 1990-2021.


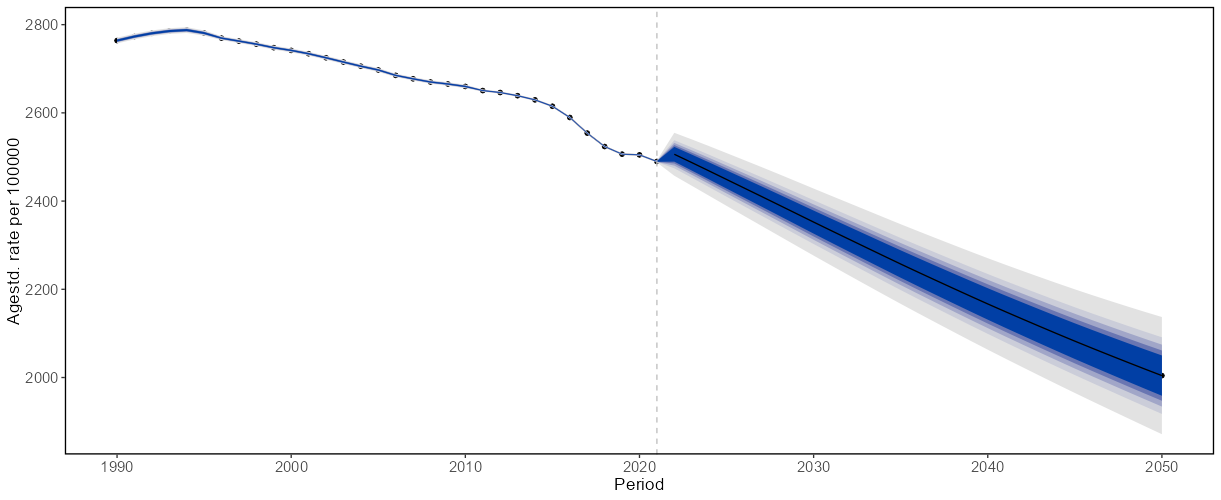


Figure S9: Projected Age-Standardized Prevalence Rates of Chronic Obstructive Pulmonary Disease in the Overall Population of China, 2022-2050.


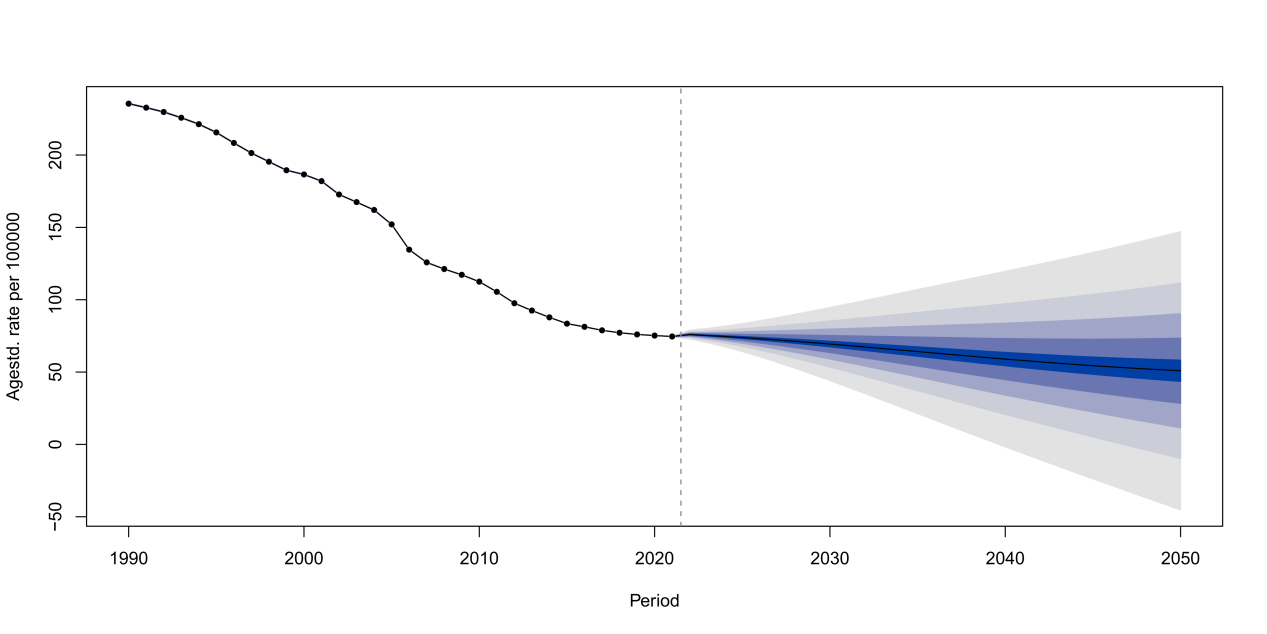


Figure S10: Projected Age-Standardized Mortality Rates of Chronic Obstructive Pulmonary Disease in the Overall Population of China, 2022-2050.


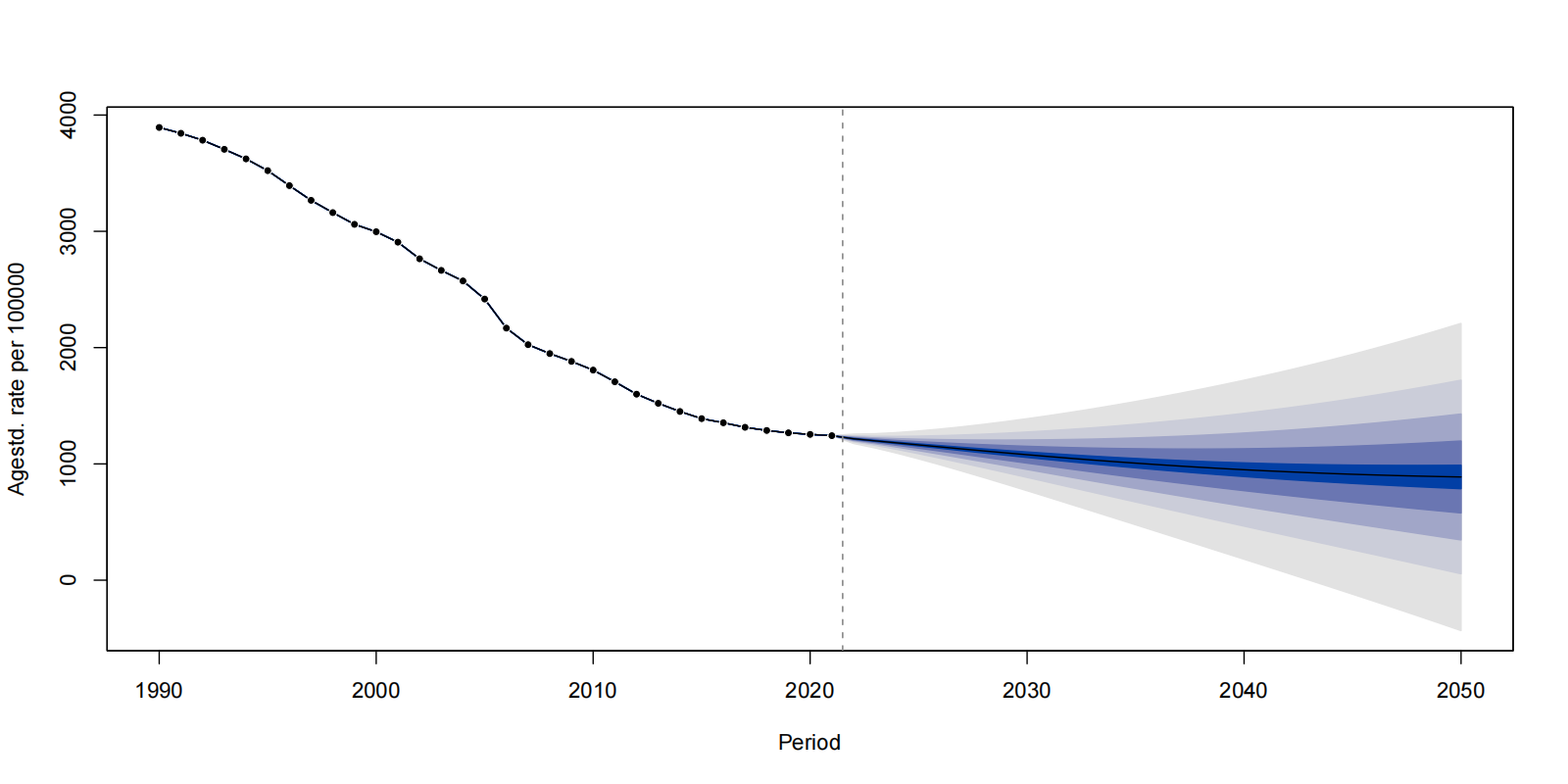


Figure S11: Projected Age-Standardized DALYs Rates of Chronic Obstructive Pulmonary Disease in the Overall Population of China, 2022-2050.
